# Fertilizer Rate-Associated Increase in Foliar Jasmonate Burst Observed in Wounded *Arabidopsis thaliana* Leaves is Attenuated at eCO_2_

**DOI:** 10.3389/fpls.2019.01636

**Published:** 2020-01-16

**Authors:** Julian Martinez Henao, Louis Erik Demers, Katharina Grosser, Andreas Schedl, Nicole M. van Dam, Jacqueline C. Bede

**Affiliations:** ^1^Department of Plant Science, McGill University, Ste-Anne-de-Bellevue, QC, Canada; ^2^German Centre for Integrative Biodiversity Research (iDiv) Halle-Jena-Leipzig, Friedrich-Schiller-University Jena, Leipzig, Germany

**Keywords:** ascorbate, carbon dioxide, glutathione, jasmonate, nitrogen fertilizer, oxidative stress, pyridine nucleotides, wounding

## Abstract

The predicted future increase in tropospheric carbon dioxide (CO_2_) levels will have major effects on C_3_ plants and their interactions with other organisms in the biosphere. In response to attack by chewing arthropod herbivores or nectrotrophic pathogens, many plants mount a rapid and intense increase in jasmonate-related phytohormones that results in a robust defense response; however, previous studies have shown that C_3_ plants grown at elevated CO_2_ may have lower induced jasmonate levels, particularly in well nitrate-fertilized plants. Given the relationship between atmospheric CO_2_, photorespiration, cellular reductant and redox status, nitrogen assimilation and phytohormones, we compared wound-induced responses of the C_3_ plant *Arabidopsis thaliana*. These plants were fertilized at two different rates (1 or 10 mM) with nitrate or ammonium and grown at ambient or elevated CO_2_. In response to artificial wounding, an increase in cellular oxidative status leads to a strong increase in jasmonate phytohormones. At ambient CO_2_, increased oxidative state of nitrate-fertilized plants leads to a robust 7-*iso*-jasmonyl-L-isoleucine increase; however, the strong fertilizer rate-associated increase is alleviated in plants grown at elevated CO_2_. As well, the changes in ascorbate in response to wounding and wound-induced salicylic acid levels may also contribute to the suppression of the jasmonate burst. Understanding the mechanism underlying the attenuation of the jasmonate burst at elevated CO_2_ has important implications for fertilization strategies under future predicted climatic conditions.

## Introduction

Climate change, in particular increasing tropospheric carbon dioxide (CO_2_) levels, will bring challenges to agriculture and forestry in terms of plant resistance to biotic stresses, such as pathogens and insect herbivores (Zavala et al., 2013; Zavala et al., 2017; Pincebourde et al., 2017; Kazan, 2018). To date, research on plant responses to biotic stresses under elevated CO_2_ have been somewhat contradictory which reflects our lack of understanding of these complex interactions and the integration of factors involved in the regulation of plant defenses. Yet, for global food production and security, it is imperative to understand how plant defense responses will change. This will allow us to make appropriate decisions in these rapidly changing environmental conditions.

In the last 50 years, atmospheric CO_2_ levels have increased more than 20% from 322 to >410 ppm (https://www.esrl.noaa.gov/gmd/ccgg/trends/). These levels are predicted to reach between 600 to 1,000 ppm by the end of this century (Cox et al., 2000; Intergovernmental Panel on Climate Change, 2013; www.ipcc-data.org/observ/ddc_co2.html). On the face of it, one might imagine an increase in plant productivity, particularly of C_3_ plants. In C_3_ plants, increased atmospheric CO_2_ levels will result in more efficient photosynthesis by reducing flux through the C2 photorespiration pathway (Long et al., 2004; Ehlers et al., 2015). However, this has not always shown to be the case, emphasizing that plant productivity reflects complex interactions between carbon and nitrogen metabolism that also are linked to cellular redox status and stress signaling (Foyer et al., 2006; Long et al., 2006; Foyer et al., 2009; Foyer and Noctor, 2011; Foyer et al., 2011; Noctor et al., 2012; Noctor and Mhamdi, 2017).

**Figure 1:**
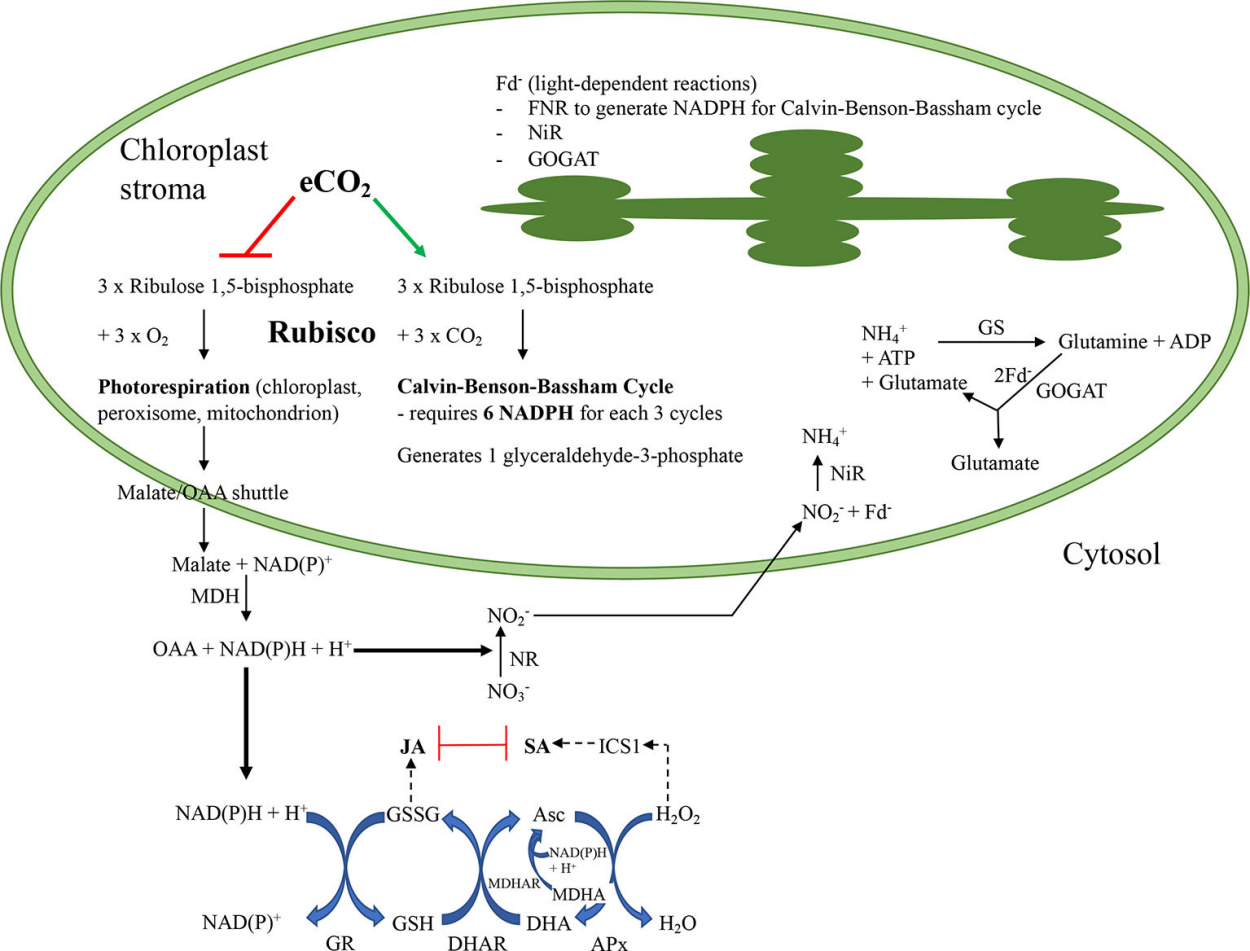
Summary of putative interactions as atmospheric carbon dioxide levels increase in a C3 plant that assimilates nitrate in their leaves. At present atmospheric conditions, the enzyme ribulose 1,5-bisphosphate carboxylase/oxygenase (Rubisco) catalyzes both the carboxylation of ribulose 1,5-bisphosphate, utilizing ATP and NADPH + H^+^ to generate triose-phosphates through the Calvin-Benson-Bassham (CBB) cycle that can be used in sucrose biosynthesis, or the oxygenation, to produce compounds that must be “salvaged” through photorespiration (PR). In the PR pathway, reductant equivalents are shuttled from the chloroplast through the malate/oxaloacetic acid (OAA) transporter to the cytosol and then peroxisome. A portion of these reductant equivalents remain in the cytosol where they are used for metabolic processes, such as to maintain redox homeostasis through the glutathione-ascorbate Foyer-Halliwell-Asada cycle or nitrate reduction. After nitrate is reduced, nitrite is transported into the chloroplast where it is reduced by nitrite reductase (NiR) and reduced ferredoxin (Fd^-^) to ammonium ion. Ammonium ion, either produced from nitrate assimilation or received *via* the phloem, is further converted to glutamine and then glutamate; this later conversion, catalyzed by glutamine-*oxo*glutarate aminotransferase (GOGAT) also requires Fd^-^. Thus, Fd^-^ produced through the thylakoid-associated light-dependent reactions is necessary for nitrogen assimilation and also to generate NADPH required for the CBB cycle *via* the Fd^–^dependent NADP^+^ reductase (FNR). Under predicted future environmental conditions, elevated atmospheric carbon dioxide (eCO_2_) will favour the Rubisco carboxylation over oxygenation reaction, increasing the demand for NADPH, generated by FNR, in the CBB cycle. Cytosolic reductant levels may decrease as photorespiration decreases and this may affect cellular redox balance (Foyer-Halliwell-Asada cycle) that links to defence phytohormone levels as well as nitrate assimilation. The phytohormones jasmonic acid (JA) and salicylic acid (SA) have reciprocal negative effects on each other which allows the plant to fine tune its defense responses to the environment. Asc, reduced form of ascorbate; CBB, Calvin-Benson-Bassham cycle; DHA, dehydroascorbate, oxidized form of ascorbate; eCO_2_, elevated carbon dioxide; Fd^-^, reduced ferredoxin; FNR, ferredoxin-dependent NADPH reductase; GS, glutamine synthetase; GSH, reduced glutathione; GOGAT, glutamine-*oxo*glutarate aminotransferase; GR, glutathione reductase; GSSG, oxidized glutathione; H_2_O, water; H_2_O_2_, hydrogen peroxide; ICS1, isochorismate synthase1; JA, jasmonic acid; MDH, malate dehydrogenase; MDHA, monodehydroascorbate; MDHAR, monodehydroascorbate reductase; NAD, oxidized form of nicotinamide adenine nucleotide; NADH, reduced form of nicotinamide adenine nucleotide; NADP, oxidized form of nicotinamide adenine nucleotide phosphate; NADPH, reduced form of nicotinamide adenine nucleotide phosphate; NH_4_^+^, ammonium ion; NiR, nitrite reductase; NO_2_^-^, nitrite; NO_3_^-^, nitrate; NR, nitrate reductase; OAA, oxaloacetic acid; PR, photorespiration; Rubisco, ribulose 1,5-bisphosphate carboxylase/oxygenase; SA, salicylic acid.

Ribulose 1,5-bisphosphate carboxylase/oxygenase (Rubisco), one of the most abundant foliar enzymes, plays a key role in photosynthesis by fixing CO_2_ to the 5C substrate, ribulose 1,5-bisphosphate in the Calvin-Benson-Bassham (CBB) cycle (**Figure 1**) (Carmo-Silva et al., 2015). However, if oxygen is fixed instead of CO_2_, then the resultant 2C phosphoglycolate must be salvaged through the C2 photorespiration pathway. Traditionally, the photorespiration pathway was viewed as wasteful as it requires ATP and NAD(P)H to regenerate a metabolically useful triose-phosphate and removes metabolic intermediates from the CBB cycle, thereby limiting the rate of this cycle and net photosynthesis by at least 25% (Ehlers et al., 2015). However, this view is rapidly changing as the importance of this pathway in integrating physiological process such as photosynthesis, nitrogen assimilation through redox balance and signaling is now recognized (Betti et al., 2016; Eisenhut et al., 2019).

As atmospheric CO_2_ levels increase, Rubisco will be more efficient at fixing CO_2_ over O_2_ and flux through the photorespiration pathway will be reduced. Therefore, in many C_3_ plant species, exposure to elevated CO_2_ (eCO_2_) levels results initially in an enhanced photosynthetic efficiency which often returns to basal levels over time, a process known as acclimation (Stitt and Krapp, 1999; Rogers and Humphries, 2000; Ainsworth and Rogers, 2007). A hallmark of photosynthetic acclimation is a decrease in plant nitrogen concentration, often resulting from a downregulation of Rubisco levels and activity, that act to lower the photosynthetic rate (Long et al., 2004). A number of factors contribute to this eCO_2_-associated adjustment of foliar nitrogen levels, including carbohydrate-mediated repression of genes that encode photosynthetic proteins as well as inhibition of nitrate assimilation (Leakey et al., 2009; Kant et al., 2012; Bloom, 2015a).

Nitrate assimilation is reported to be negatively impacted by eCO_2_ in many plant species, particularly C_3_ plants that assimilate nitrate in their leaves (Bloom, 2015a; Bloom, 2015b). The conversion of nitrate to organic nitrogen is energy intensive and begins with the NAD(P)H-dependent reduction of nitrate by nitrate reductase to produce the highly reactive, toxic nitrite that is rapidly transported into the plastid (Xu et al., 2012; Krapp, 2015; Huma et al., 2018). In photosynthetic tissue, nitrite reductase reduces nitrite to ammonium ion using reduced ferrodoxin (Fd^-^), generated through the light-dependent reactions, as the electron donor. Therefore, when comparing the energy requirements of different fertilizer nitrogen sources, the use of nitrate fertilization costs an additional 8 e^-^/mole nitrogen compared to the direct use of ammonium fertilizer (Williams et al., 1987; Noctor and Foyer, 1998; Bloom, 2015b).

At eCO_2_, nitrate assimilation is impaired in the monocot wheat and the dicot *Arabidopsis thaliana* when plants were grown in growth chambers or wheat in the field under eCO_2_ conditions (Bloom et al., 2010; Bloom et al., 2014). Under eCO_2_ or elevated O_2_, both conditions that reduce photorespiration, a reduction in shoot nitrate assimilation was observed (Rachmilevitch et al., 2004; Bloom et al., 2010; Bloom 2015a), indicating a strong linkage between photorespiration and nitrate metabolism. Malate export from the chloroplast to the cytosol, that is linked to the photorespiration pathway, provides an important NADH source for nitrate reductase, therefore, under eCO_2_ with the suppression of photorespiration, a decrease in cytosolic reducing power could negatively affect nitrate reduction (Scheibe, 2004; Scheibe et al., 2005; Bloom, 2015b). However, even in the night, eCO_2_ inhibits nitrate, but not ammonium, assimilation (Rubio Asensio et al., 2015); therefore, other mechanisms, in addition to a link to photorespiration, must also be involved. At night, eCO_2_ may negatively affect mitochondrial pathways to decrease energy supply. In both the day or night times, eCO_2_ may also inhibit translocation of nitrite into the chloroplast (Bloom et al., 2002), though this seems unlikely given its toxicity. During the day, reduction of nitrite to ammonium in the chloroplast requires electrons from reduced Fd^-^. Therefore, there will be competition for reduced Fd between the Fd-dependent NADPH reductase (FNR), nitrite reductase and glutamine-*oxo*gluterate aminotransferase with FNR having the highest affinity for reduced Fd^-^. Therefore, at eCO_2_, as photosynthetic efficiency increases, stromal NADPH will be synthesized and used in the CBB cycle, at the expense of other pathways, such as nitrogen assimilation (Baysdorfer and Robinson, 1985; Huma et al., 2018). Though this is contentious and other studies have observed that atmospheric CO_2_ levels did not affect nitrogen assimilation in *Phaseolus vulgaris* or wheat (Andrews et al., 2019). Photorespiration and nitrate assimilation also increase the rate of plant CO_2_ uptake (Busch et al., 2018); CO_2_ uptake declined when nitrate-fertilized plants were grown under conditions that limited photorespiration.

eCO2 also affects the foliar pyridine nucleotide (NAD^+^/NADH, NADP^+^/NADPH) and redox (ascorbate, glutathione) pools that link important plant physiological pathways, such as seed germination, stomatal regulation, vegetative-to-reproductive transition and stress and defense responses (**Figure 1**) (Noctor et al., 2012; Considine and Foyer 2014; Noctor and Mhamdi, 2017; Igamberdiev and Bykova, 2018). Therefore, perturbation of the cellular pyridine nucleotide and redox status in plants grown at eCO_2_ may affect the plant’s ability to respond to biotic stresses, such as pathogens or insect herbivores. The Foyer–Halliwell–Asada cycle is an interconnected series of reduction and oxidation (redox) reactions of glutathione and ascorbate that uses NAD(P)H as the final reductant (Foyer and Noctor, 2011; Noctor et al., 2012). This node integrates and translates plant metabolic status and environmental cues to changes in the cellular oxidative status to allow the plant to respond appropriately to the everchanging environment (Potters et al., 2010; Noctor et al., 2011). Dynamic changes cellular reductant (total and ratio oxidized/reduced pyridine nucleotides) and redox balance (total and oxidized/reduced ascorbate and glutathione pools) act to signal downstream molecular and biochemical responses. The photorespiration pathway provides input into the cellular redox balance by the generation of the reactive species hydrogen peroxide (H_2_O_2_) from the peroxisome and through the export of malate from chloroplasts into the cytosol to generate NADH (Scheibe, 2004; Scheibe and Dietz, 2012). The eCO_2_-associated reduction in photorespiration will lead to lower peroxisomal H_2_O_2_ production that should translate into a more highly reduced cellular environment (Foyer et al., 2009; Bloom 2015a; Bloom, 2015b). However, in *Arabidopsis* grown at eCO2, there was activation of signaling pathways that reflected a more oxidative state (Mhamdi et al., 2013; Mhamdi and Noctor, 2016). This likely reflects a reduction in the malate shuttle and, hence, a decrease in cytosolic NAD(P)H as photorespiration decreases in plants grown at eCO_2_ (Selinski and Scheibe, 2019). It also must be recognized that glutathione may also be linked to oxidative signaling independently of ascorbate (the Foyer–Halliwell–Asada cycle) through glutaredoxin–periredoxin or glutathione/thioredoxin peroxidase pathways (Noctor et al., 2012).

Developmental and environmental cues are integrated and reflected in the cellular redox balance that then signals the appropriate response through the actions of phytohormones (Noctor et al., 2011; Han et al., 2013a). Though phytohormones generally lead to specific types of downstream action, for example salicylic acid (SA) is involved in plant defense against biotrophic pathogens and 7-jasmonyl-isoleucine (JA-Ile) is involved in plant defense against chewing insect herbivores, these hormones often modify each other’s actions through signaling nodes; a phenomenon referred to as “phytohormone cross-talk” (Koornneef and Pieterse, 2008; Caarls et al., 2015). Thus, SA and jasmonates often act antagonistically to modify the final response allowing the plant to integrate multiple developmental and environmental cues to respond appropriately (Caarls et al., 2015). Strong evidence is emerging for critical links between redox metabolites, in particular glutathione, and phytohormone signaling (Mhamdi et al., 2013; Han et al., 2013a; Han et al., 2013b).

Damage to leaves by mechanical wounding or chewing insect herbivores, such as caterpillars and beetles, activates signaling pathways that cause a rapid and vigorous increase in octadecanoid-derived phytohormones, collectively called jasmonates, that lead to induction of downstream defense responses (Arimura et al., 2011; Wasternack and Hause, 2013; Howe et al., 2018). The cellular state of oxidized glutathione (GSSG and GSSG/total glutathione) is highly correlated with expression of jasmonate-dependent genes, such as *AtLOX3, AtMYB95, AtJAZ10*, and *AtVSP2* (Mhamdi et al., 2010; Gfeller et al., 2011; Han et al., 2013a); therefore, oxidative stress in response to damage may link to jasmonate levels and dependent gene expression. Glutathione regulates both the nonexpressor of pathogenesis-related protein1 (NPR1)-dependent and -independent SA signaling pathways (Han et al., 2013b; Kovacs et al., 2015). In recognition of biotrophic pathogens, a feedforward escalation between a SA and H_2_O_2_ loop leads to changes in the cellular redox status to activate NPR1, which is regulated by through reduction of disulfide bridges regulated by H-type thioredoxins to lead to SA-dependent gene expression (Spoel et al., 2003; Koornneef et al., 2008; Tada et al., 2008). As well, an oxidative glutathione redox status is implicated in moderating the SA x jasmonate crosstalk but through an NPR1-independent, isochorismate synthase1 (ICS1)-dependent mechanism that is still not completely understood (Mhamdi et al., 2010; Han et al., 2013a; Han et al., 2013b).

In general, plants grown under eCO_2_ have a higher cellular oxidative state resulting in increased foliar SA accumulation (Casteel et al., 2012a; Casteel et al., 2012b; Zavala et al., 2013; Mhamdi et al., 2013; Mhamdi and Noctor 2016; Gog et al., 2019). In *Arabidopsis*, eCO_2_ did not affect redox ratios but total glutathione and NAPDH pools were increased. This eCO_2_-associated increase in SA often translates into enhanced protection against biotrophic pathogens (Mhamdi and Noctor, 2016; Noctor and Mhamdi, 2017; Williams et al., 2018). However, this is not always the case, resistance to *Pseudomonas syringae* pv. *tomato* infection was compromised in *Arabidopsis* grown at eCO_2_ (Zhou et al., 2019), pointing to the involvement of additional factors such as day length and nitrogen fertilization. In comparison, jasmonate levels are often suppressed in plants grown at eCO_2_ leading to a higher susceptibility to chewing insect herbivores (Casteel et al., 2008; Zavala et al., 2013; Gog et al., 2019). Again, jasmonate levels at eCO_2_ appear to be variable across different experiments, pointing to differences in plant species or involvement of other environmental factors, particularly light photoperiod and/or intensity (Matros et al., 2006; Casteel et al., 2012a; Casteel et al., 2012b; Zavala et al., 2013; Mhamdi and Noctor, 2016; Gog et al., 2019). Paudel et al. (2016) found that the foliar jasmonate burst that leads to induced resistance against chewing insect herbivores is attenuated in wounded plants grown at eCO_2_ under high nitrate fertilization. Therefore, nitrogen may play a role in the plant’s response to wounding stress at eCO_2_.

Given the relationship between eCO_2_, photorespiration, nitrate assimilation and cellular redox status and phytohormones that mediate plant defense responses, we conducted this study to compare responses to foliar wounding in *Arabidopsis* that were fertilized at two different rates (1 or 10 mM) of nitrate or ammonium in plants grown at aCO2 or eCO2. *A. thaliana* is a C_3_ plant that assimilates a significant proportion of nitrate in the leaves, compared to the roots (Kalcsits and Guy, 2013). In plants grown at eCO_2_, if the resultant lower photorespiration alters reductant availability necessary for nitrogen assimilation, then this should be reflected in pyridine nucleotide ratios and levels (**Figure 1**). Lower pyridine nucleotide levels and/or changed oxidized-to-reduced ratios will affect cellular redox metabolites levels and ratios that may then be reflected in phytohormone levels. Therefore, through this research, we seek to further understand the mechanism underlying the suppression of the jasmonate burst in response to wounding of foliar tissues in well nitrate-fertilized *Arabidopsis* plants grown at eCO2 (Paudel et al., 2016).

## Materials and Methods

### Plants

*A. thaliana* Col-0 (TAIR CS3749) seeds, that were surface-sterilized in 3% (v/v) NaOCl for 10 min, followed by a rinse in 70% EtOH for 1 min and several washes in sterile ddH_2_O, were placed in Petri dishes containing Murashige and Skoog nutrient solution, 1% agar (Boyes et al., 2001). After cold treatment at 4**°**C for 72 h in the dark to break seed dormancy, plates were maintained at 20 ± 1**°**C, 60–70% relative humidity in continuous light (light intensity 250 μE m^-2^ s^-1^, 23**°**C).

### Experimental Design

Plants with two true leaves were transferred into individual square pots (6.6 × 6.6 × 9 cm^3^) containing Farfard Agromix PV20 potting medium and randomly placed in in the growth cabinet (12 h light at 22**°**C with light intensity ramped to 250 μE m^-2^ s^-1^ over 3 h:12 h dark at 18**°**C ramped over 3 h) with controlled CO_2_ concentrations, either 450 ± 50 or 900 ± 50 ppm. Plants were fertilized three times a week with half strength modified Hoagland’s solution (macronutrients: 0.5 mM KH_2_PO_4_, 0.5 mM CaCl_2_, 0.25 mM MgSO_4_, 0.05 mM Na_2_EDTAFE^III^; micronutrients: 46 μM H_3_BO_3_, 9 μM MnSO_4_, 0.32 μM CuSO_4_·5H_2_0, 0.76 μM Zn SO_4_·H_2_O, 0.16 μM Na_2_MoO_4_·2H_2_O, 0.002 μM CoCl_2_·6H_2_O) with either KNO_3_ (1 or 10 mM) or (NH_4_)_2_SO_4_ (1 or 10 mM) as the nitrogen source. Nitrogen treatment was randomized. Ammonium is toxic to many plant species when present in excess (Li et al., 2014). *Arabidopsis* is negatively affected by 5-mM ammonium when grown hydroponically (Podgórska et al., 2015). At the time of our experiment, we did not visually observe a difference between plants fertilized with nitrate or ammonium that likely reflects that our plants were grown in potting media.

When plants were 4 weeks old (stage 3.9; Boyes et al., 2001), a subset of plants were frozen in liquid nitrogen (N_2_) to measure foliar carbon (C) and nitrogen (N) levels. Half of the remaining plants were wounded on the largest six rosette leaves with an hole punch, carefully ensuring that the midvein was not damaged, at 5 pm (1.5 h before end of the day). A plexiglass panel was used to separate wounded and unwounded plants to minimize any volatile signaling between plants (Lan et al., 2014). For the measurement of pyridine nucleotides and redox metabolites, whole rosettes were immediately frozen in N_2_ over a time course (15, 30, and 45 min). Rosettes for phytohormone analyses were taken 24 h after mechanical damage and immediately flash-frozen in N_2_ and stored at -80°C until analysis. The experiment was temporally repeated at least four times with one biological replicate being sampled at each independent replication (total n = 4).

### Nutrient Controls

Plants were fertilized with different nitrogen sources, either nitrate (NO_3_^-^) or ammonium (NH_4_^+^), that represent an anion or cation. Therefore, controls must be conducted to ensure that the differences in the counterion (K^+^ for nitrate and SO_4_^-^ for ammonium ion) do not influence experimental results (Vatter et al., 2015). Therefore, controls of the addition of 9.2 or 9 mM K_2_SO_4_ to either 1 mM of nitrate or ammonium fertilizer were used. Osmolality and osmotic pressure were calculated according to Trejo-Téllez and Gómez-Merino (2012) (**Supplemental Table 1**).

### Carbon and Nitrogen Quantification

*Arabidopsis* foliar carbon and nitrogen levels were measured by a CN elemental analyzer (Vario EL cube analyzer, Elementar Analysensysteme). Lyophilized samples were ground using a sonicator (Qiagen TissueLyser II). Then after transport to Germany, re-dried in an oven at 70°C for 2 h. Samples were weighed into tin boats (4 × 4 × 11 mm^3^, Elementar Analysensysteme) using an analytical microbalance (Cubis MSA, Sartorius AG). The tin boats were closed tightly over the samples and sealed to minimize air infiltration. The final pellet was reweighed and stored in a dessicator until measurement. Elemental carbon and nitrogen were quantified following manufacturer’s protocol.

### Pyridine Nucleotide Quantification

Pyridine nucleotide (NAD^+^, NADH, NADP^+^, NADPH) levels were measured based on an enzymatic cycling method, with alcohol dehydrogenase to determine the NAD^+^ pool and glucose 6-phosphate dehydrogenase (G6PDH) to determine the NADH^+^ pool, which is visualized through the reduction of dichlorophenolindophenol by phenazine methosulfate (Quevel and Noctor, 2007; Noctor et al., 2016). Carefully weighed frozen leaf material (approximately 100 mg) was finely ground in liquid nitrogen. An acid extraction (cold 0.2 M HCl) was used to prepare samples for NAD^+^ or NADP^+^ analysis whereas tissue was extracted in cold 0.2 M NaOH for NADH or NADPH analysis. To remove cellular debris, samples were centrifuged at 16,000 *g* for 10 min, 4°C and the supernatant transferred to a sterile tube. Samples (0.2 ml) were boiled for 1 min and then placed immediately on ice. Phosphate buffer (0.2 M, pH 5.6, 35 ul) was added and the samples were neutralized by carefully and the samples were neutralized by carefully adding small volumes of 0.2 N NaOH to the acidic extracts (NAD^+^ or NADP^+^) extracts with vortexing until pH 6-7 was reached whereas for the basic extracts (NADH or NADPH), small volumes of 0.2 N HCl were carefully added with vortexing until a neutral pH was reached. The added volume was noted and taken into account in the final calculations. To determine NAD^+^ and NADH levels, 20 μl of the neutralized acidic (NAD^+^) or neutralized basic (NADH) extract was added to a reaction mixture with a final concentration of 50 mM HEPES buffer containing 1 mM EDTA, pH 7.5, 12 μM 2,6-dichlorophenolindophenol, 1 mM phenazine methosulfate and 125 U alcohol dehydrogenase in triplicate in a 96-well plate. After addition of ethanol (final concentration 7.4%), the change in absorbance at 600 nm was measured at 30-s intervals for 5 min using a Tecan Infinite M200 spectrophotometer. For NADP^+^ and NADPH^+^, the same general reaction mixture was used except that the substrate is 0.5 mM glucose-6-phosphate, instead of ethanol, and the enzyme is 0.01 U G6PDH, instead of alcohol dehydrogenase. The linear reaction rate is used to calculate the concentration by comparison to a standard curve of the respective pyridine nucleotide (ranging from 0 to 0.2 μM) performed in triplicate.

### Redox Metabolite Quantification

Redox metabolite (Asc, DHA, GSH, GSSG) levels were measured based on an enzymatic cycling method (Quevel and Noctor, 2007; Noctor et al., 2016). Carefully weighed frozen leaf material (approximately 100 mg) was finely ground in liquid nitrogen and extracted in cold 0.2 M HCl. To remove cellular debris, samples were centrifuged at 16,000 *g* for 10 min, 4°C. Phosphate buffer (0.2 M, pH 5.6, 0.5 ml) was added and the extracts brought to pH 4–5 by carefully adding small volumes of 0.2 N NaOH with vortexing. The added volume was noted and taken into account in the final calculations.

To determine the total glutathione pool, in triplicate in a 96-well plate, 10 μl of the neutralized extract was added to a reaction mixture where the final concentration was 0.1 M phosphate buffer containing 5 mM EDTA, pH 7.5, 0.5 mM NADPH and 0.6 mM 5,5 dithiobis 2-nitro-benzoic acid, pH 7.5. After shaking, the reaction was initiated by the addition of glutathione reductase (final concentration 0.01 U). The reaction was monitored at 412 nm for 2 min at 5 s intervals and the slope of the line from the first 90 s was used to determine total glutathione concentration, using a standard curve of free GSH (ranging from 0 to 250 pmol). To measure GSSG, the neutralized plant extracts as well as a concentration range of GSSG standards (0–100 pmol) were incubated with 2-vinylpyridine, which forms an insoluble precipitate with GSH. After centrifugation at 16,000 *g* for 10 min, room temperature, GSSG concentration was determined by the assay as described above. Reduced GSH was calculated by subtracting 2 × GSSG from the total GSH.

To determine reduced ascorbate (Asc) levels, in triplicate in a 96-well plate, 40 μl of the neutralized extract was added to a reaction mixture of 0.1 M phosphate buffer, pH 5.6 (final concentration). After shaking, the absorbance was read at 265 nm. The reaction initiated by the addition of 0.2 U of ascorbate oxidase, prepared in 0.2 M phosphate buffer, and incubated with gentle shaking for 8 min at room temperature. Again, the absorbance was read at 265 nm and the difference used to calculate Asc levels based on an Asc standard curve (ranging from 40 to 240 μM) that was included on each plate. In parallel, 100 μl of extract was added to a reaction mixture with a final concentration of 0.1 M phosphate buffer, pH 5.6 and 1.25 mM dithiothreitol, that reduces dehydroascorbate to Asc. After incubation for 30 min at room temperature, total ascorbate levels were measured.

### Phytohormone Quantification

Phytohormone analysis of *Arabidopsis* rosette tissues was conducted by ultrahigh performance liquid chromatography-triple quadrupole mass spectrometry (UHPLC-EVOQ-TQ-MS, Bruker). Homogenized, lyophilized foliar samples (approximately 20 mg) were extracted in ethyl acetate (MS-grade, VWR) containing a mixture of isotopically-labeled standards of the acidic phytohormones (D6-JA and D6-JA-Ile (HPC Standards) and D4-SA and D6-ABA (OlChemIm. s.r.o.). After vortexing for 10 min, cellular debris was removed by centrifugation at 18,994 *g* for 10 min, 4°C. The supernatant was transferred to a new tube and the solvent evaporated using a vacuum concentrator at room temperature. The pellet was resuspended in 70% methanol (v/v) and sonicated to fully solubilize the pellet and again centrifuged for 5 min at 18,994 *g*, 4°C.

Samples were separated by reverse phase UHPLC on a Zorbax Extend-C_18_ column (4.6 × 50 mm, 1.8 μm, Agilent). For the first 30 s, the mobile phase was stationary at 5% ACN, 0.05% formic acid, then this was increased to 50% ACN, 0.05% formic acid over 2 min. The mobile phase was kept at 100% ACN, 0.05% formic acid for 1 min and then return to the initial conditions over the next minute. All solvents used were liquid chromatography–mass spectrometry grade. The column temperature was 42°C.

After separation, compounds were nebulized by electron spray ionization in the negative mode using the following conditions: capillary voltage 4,500, cone 35 arbitrary units (a.u.)/350°C, probe 60 a.u./475°C, nebulizer gas (N_2_) 60 a.u. Phytohormones were identified based on retention time and monitoring the transition m/z (**Supplemental Table 2**). Phytohormone level was calculated based on the peak area of the corresponding internal standard and initial weight.

### Statistics

Foliar carbon, nitrogen and their ratio were analyzed by three-factor analysis-of-variance (ANOVA) (factors: CO_2_, nitrogen source, nitrogen level) using the statistical software SPSS (ver. 16). Reducing power and redox metabolite levels were compared at 15, 30, and 45 min after mechanical wounding by four-factor ANOVA (factors: CO_2_, nitrogen source, nitrogen level, treatment). Changes in response to mechanical damage often reflected the time after wounding. Effects of CO_2_, nitrogen source and nitrogen level were only considered significant if consistent across the time points. OPDA and JA are intermediates to biologically active form of JA-Ile (Wasternack and Hause, 2013; Howe et al., 2018). Given this relationship, the effect of CO_2_, nitrogen source, nitrogen level, and wounding on phytohormone levels was analyzed by four factor-multiple ANOVA (MANOVA). SA and ABA levels were analyzed by four-factor ANOVA. In all analyses, outliers were identified by the maximal normed residual test (Grubb’s test) (Stefanski, 1971).

In all analyses, normalization was performed if data did not follow a normal distribution and violated Levene’s test of homogeneity. The square root of OPDA and JA phytohormone data were used. The log_10_ of ratios, JA-Ile and ABA data were analyzed. Statistical differences (p ≤ 0.05) were determined by Tukey HST *post-hoc* tests.

## Results

### Counterion and Sodicity Controls

To ensure that observed effects were due to the nitrogen source and not the counterion complement of the nitrogen [*i.e.* KNO_3_ *vs* (NH_4_)_2_SO_4_] or changes in sodicity, controls to account for the potassium or sulfate imbalances were included. At either aCO_2_ or eCO2, a difference in foliar carbon (C), nitrogen (N), C:N, phytohormone, reducing agents (NAD, NADP, NADH, NADPH, and their log ratios] or redox metabolites (oxidized and reduced glutathione and ascorbate and their log ratios) was not observed (**Supplemental Table 3**).

### Foliar Carbon and Nitrogen Levels

**Figure 2:**
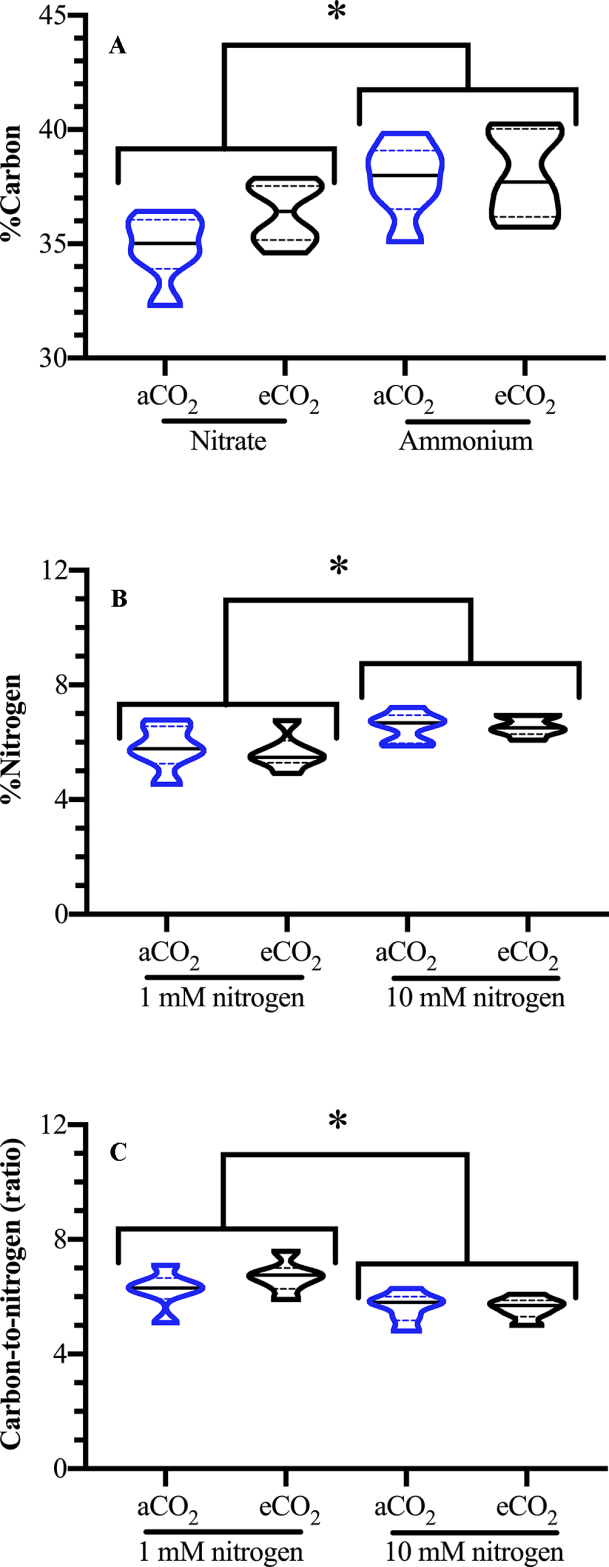
Effect of atmospheric carbon dioxide and nitrogen fertilization on *Arabidopsis* foliar carbon and nitrogen levels. *Arabidopsis thaliana* plants were grown either at ambient (aCO_2_, 450 ppm) or elevated (eCO_2_, 900 ppm) carbon dioxide and fertilized with either nitrate or ammonium ion (1 or 10 mM). Foliar **(A)** carbon (%C), **(B)** nitrogen (%N), and **(C)** ratio carbon to nitrogen (C:N); blue denotes plants grown at aCO_2_ and black denotes plants grown at eCO_2_. The experiment was repeated four times and outliers removed by the maximal normed residual test (n = 3–4). C:N ratio data was normalized by log_10_ transformation. Statistical differences were determined by three-factor ANOVA (treatments: nitrogen source, nitrogen level, CO_2_; p ≤ 0.05) followed by Tukey honestly significant difference *post hoc* test. Only significant effects are graphically represented. Data is represented by violin plots with the median indicated by a solid line and the interquartile by dashed lines. Significant differences are indicated by asterisks; * represents p ≤ 0.02.

Foliar carbon levels were significantly affected by the nitrogen source but not by nitrogen levels [Source F_(1,24)_ = 13.612; *p* = 0.002; Levels F_(1,24)_ < 0.001; *p* = 0.986]; carbon levels were elevated by 6% in ammonium-fertilized plants (**Figure 2A**). As expected, nitrogen fertilization level, but not type, affected foliar nitrogen, with an increase of leaf nitrogen in well-fertilized plants [Source F_(1,24)_ = 0.936; *p* = 0.347; Levels F_(1,24)_ =; *p* = 0.004] (**Figure 2B**). This is reflected in the foliar log C:N ratio, where well fertilized plants had a lower C:N ratio [Levels F_(1,24)_ = 10.099; *p* = 0.006] (**Figure 2C**).

Experiments were conducted in controlled climate chambers. Under these conditions, the soil may be exposed to microbes that could influence the fertilization form; for example, *Nitrosomas* and *Nitrobacter* bacteria involved in ammonia conversion to nitrate (Burger and Jackson, 2003). Even though, this is a possibility, nitrate- and ammonium ion-specific responses suggest that microbial conversion of the added fertilizer was minimal.

### Foliar Reducing Power and Redox Metabolites Reflect Nitrogen Source

**Figure 3:**
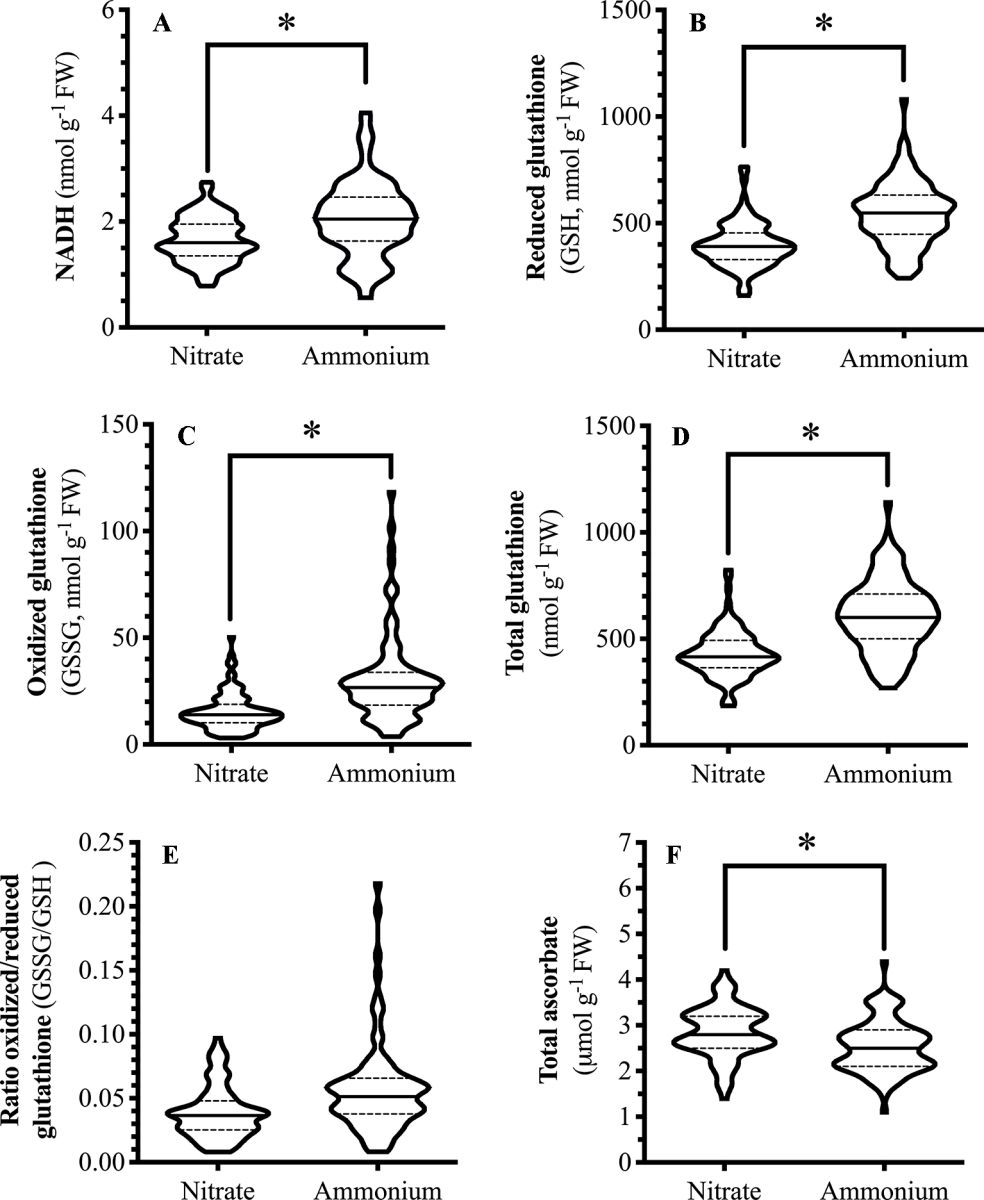
Effect of nitrogen source on Arabidopsis foliar pyridine nucleotides and redox metabolite levels. *Arabidopsis thaliana* plants were grown either at ambient (aCO_2_, 450 ppm) or elevated (eCO_2_, 900 ppm) carbon dioxide and fertilized with either nitrate or ammonium ion (1 or 10 mM). Rosette tissue of a subset of plants was mechanically damaged. Metabolite **(A)** NADH **(B)** GSH **(C)** GSSG **(D)** total glutathione **(E)** ratio GSSG/GSH **(F)** total ascorbate levels. The experiment was repeated four times and outliers removed by the maximal normed residual test (n = 3-4). GSSG/GSH ratio data was normalized by log_10_ transformation. Statistical differences were determined by four-factor ANOVA (treatments: nitrogen source, nitrogen level, CO_2_, mechanical damage; p ≤ 0.05) followed by Tukey honestly significant difference *post hoc* test. Only significant effects are graphically represented and effects were only considered significant if consistent across the time points (15, 30, 45 min.). Data is represented by violin plots with the median indicated by a solid line and the interquartile by dashed lines. Significant differences are indicated by astericks; *represents p ≤ 0.05. FW, frozen weight; GSH, reduced glutathione; GSSG, oxidized glutathione; NADH, reduced form of nicotinamide adenine nucleotide.

Foliar NADH levels varied with the nitrogen fertilizer used; NADH levels in ammonium-fertilized plants were over 20% higher than in nitrate-fertilized plants (**Figure 3A**). NAD(P)H supplies the reductant for the Foyer-Halliwell-Asada cycle (Foyer and Noctor, 2011). As foliar levels of reductant, such as NADH, increases, one would predict this to be reflected in GSH and Asc levels. Similar to NADH, GSH levels are higher in ammonium-fertilized plants compared to nitrate-fertilized plants (**Figure 3B**). GSSG and total glutathione levels are also higher in ammonium-fertilized plants (**Figures 3C**, **D**); thus, the ratio of GSH/GSSG was not affected by nitrogen treatment (**Figure 3E**). In comparison, total ascorbate levels are higher in nitrate-fertilized plants compared to those fertilized with ammonium (**Figure 3F**).

### Foliar Reducing Power and Redox Metabolite Dynamics in Wounded Leaves

**Figure 4:**
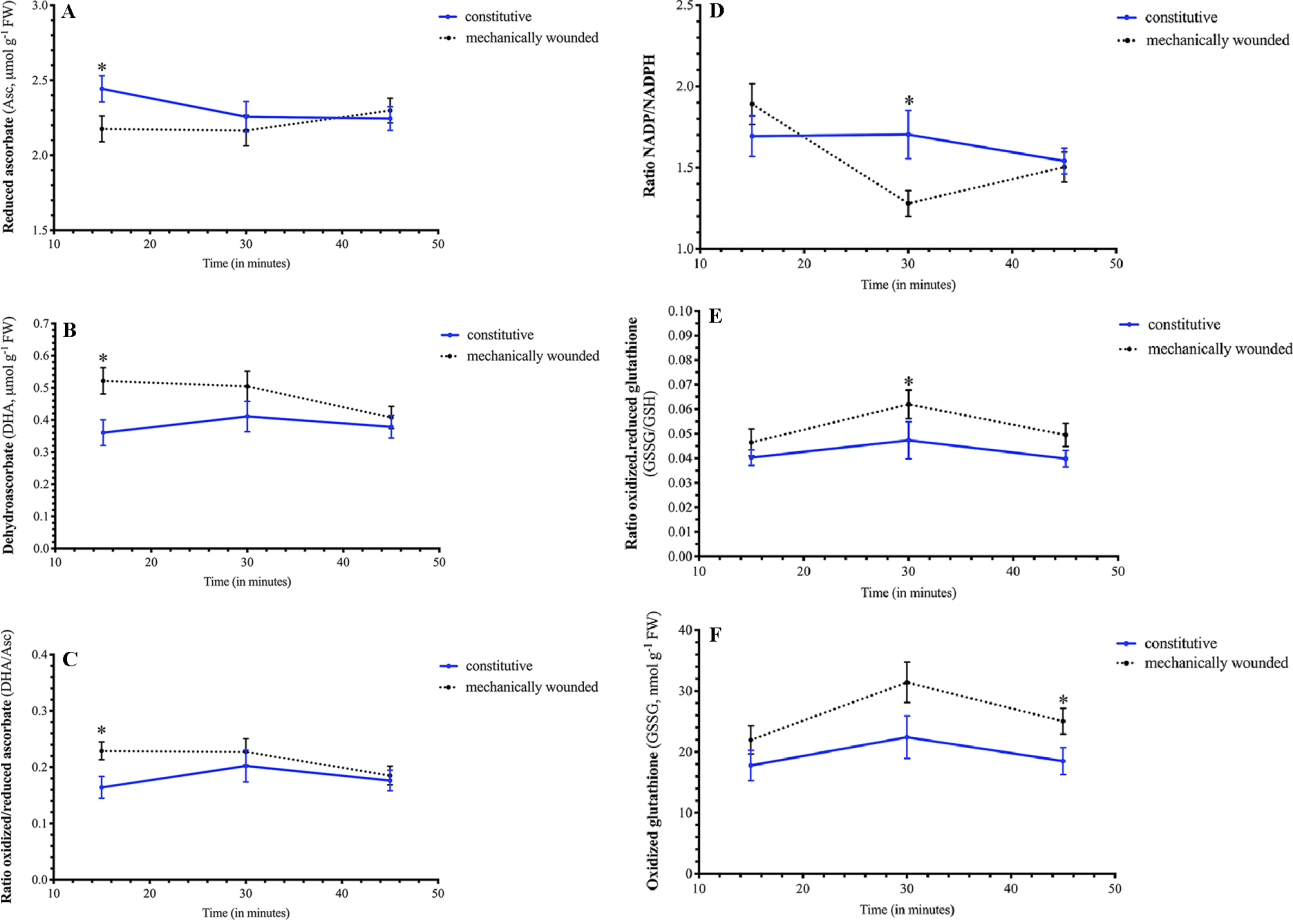
Effect of wounding on Arabidopsis foliar pyridine nucleotide and redox metabolite levels. *Arabidopsis thaliana* plants were grown either at ambient (aCO_2_, 450 ppm) or elevated (eCO_2_, 900 ppm) carbon dioxide and fertilized with either nitrate or ammonium ion (1 or 10 mM). Rosette tissue of a subset of plants was mechanically damaged. Wounding effected foliar levels of **(A)** reduced Asc, **(B)** oxidized DHA, **(C)** DHA/Asc ratio, **(D)** NADP/NADPH ratio, **(E)** GSSG/GSH ratio, and **(F)** GSSG. Blue solid lines represent constitutive levels and black dashed lines represent levels in wounded plants. The experiment was repeated four times and outliers removed by the maximal normed residual test (n = 3–4). DHA/Asc, NADP/NADPH and GSSG/GSH ratio data was normalized by log_10_ transformation. Statistical differences were determined by 4–factor ANOVA (treatments: nitrogen source, nitrogen level, CO_2_, mechanical damage; p ≤ 0.05) at each time point followed by Tukey honestly significant difference *post hoc* test. Only significant effects are graphically represented and significant differences are indicated by an asterisk; *represents p ≤ 0.05. DHA, dehydroascorbate (oxidized form of ascorbate); FW, frozen weight; GSH, reduced glutathione; GSSG, oxidized glutathione; NADP, oxidized form of nicotinamide adenine nucleotide phosphate; NADPH, reduced form of nicotinamide adenine nucleotide phosphate.

Wounding resulted in a rapid decrease in *Arabidopsis* foliar levels of reduced Asc and concomitant increase in oxidized DHA, that both return to basal levels 30 min after damage (15 min, **Figures 4A**, **B**); this translates into a wound-associated increase in the DHA/Asc ratio (**Figure 4C**). The foliar NADP/NADPH ratio rapidly decreased within 30 min after being damaged (**Figure 4D**). As well, the GSSG/GSH ratio increased in plants 30 min after wounding (**Figure 4E**). At 45 min after damage, the GSSG/GSH ratio returned to basal levels but foliar GSSG levels remained high (**Figure 4F**). Together, these metabolic changes reflect a cellular oxidative environment in response to wounding stress.

**Figure 5:**
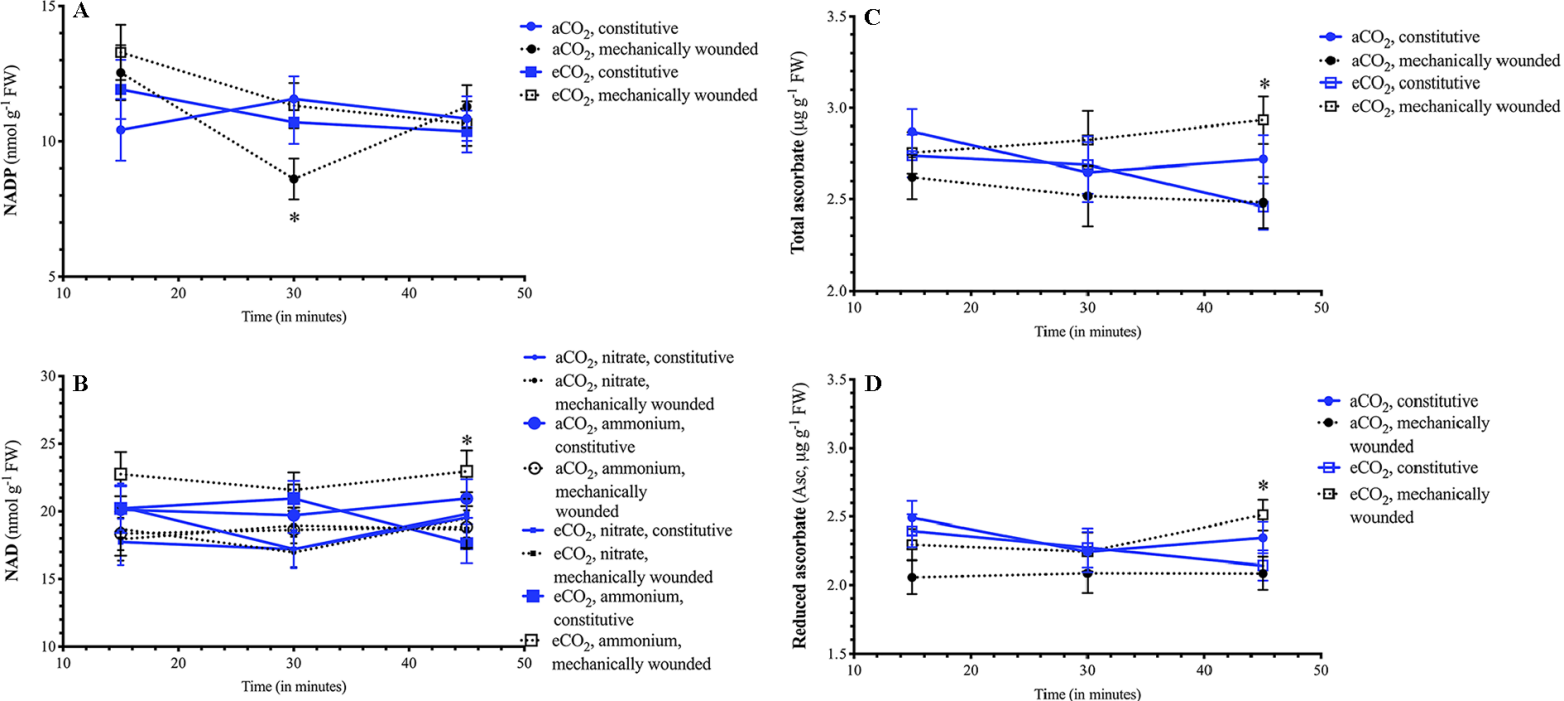
Effect of wounding on *Arabidopsis* foliar pyridine nucleotide and redox metabolite levels in plants grown at different carbon dioxide levels. *Arabidopsis thaliana* plants were grown either at ambient (aCO_2_, 450 ppm) or elevated (eCO_2_, 900 ppm) carbon dioxide and fertilized with either nitrate or ammonium ion (1 or 10 mM). Rosette tissue of a subset of plants was mechanically damaged. Depending on the atmospheric CO_2_ plants were grown in a differential effect of wounding was observed on **(A)** NADP, **(B)** NAD, **(C)** total ascorbate and **(D)** reduced Asc levels. Blue solid lines represent constitutive levels and black dashed lines represent levels in wounded plants. Circles (o) indicate plants that were grown at aCO_2_ and squares (☐) indicate plants that were grown at eCO_2_. For NAD, the size of the symbol indicates the nitrogen source (small = nitrate, large = ammonium). The experiment was repeated four times and outliers removed by the maximal normed residual test (n = 3–4). Statistical differences were determined by 4-factor ANOVA (treatments: nitrogen source, nitrogen level, CO_2_, mechanical damage; p ≤ 0.05) at each time point followed by Tukey honestly significant difference *post hoc* test. Only significant effects are graphically represented and significant differences are indicated by an asterisk; *represents p ≤ 0.05. FW, frozen weight; NAD, oxidized form of nicotinamide adenine nucleotide; NADP, oxidized form of nicotinamide adenine nucleotide phosphate.

A wound-dependent connection between pyridine nucleotide and redox status and atmospheric CO_2_ was observed. The decrease in foliar NADP levels 30 min after wounding was observed in plants grown at aCO_2_, but not in plants grown at eCO_2_ (**Figure 5A**). As well, 45 min after wounding, foliar NAD levels increased in ammonium-fertilized plants that were grown at eCO_2_ (**Figure 5B**). In plants grown at eCO_2_, total ascorbate leaf levels rapidly changed in plants within 45 min after wounding (**Figure 5C**). This primarily reflects changes in reduced Asc levels that that increase in wounded plants grown at eCO_2_ but remain stable in plants grown at aCO_2_ (**Figure 5D**).

### Foliar Phytohormone Levels

#### Jasmonates

**Figure 6:**
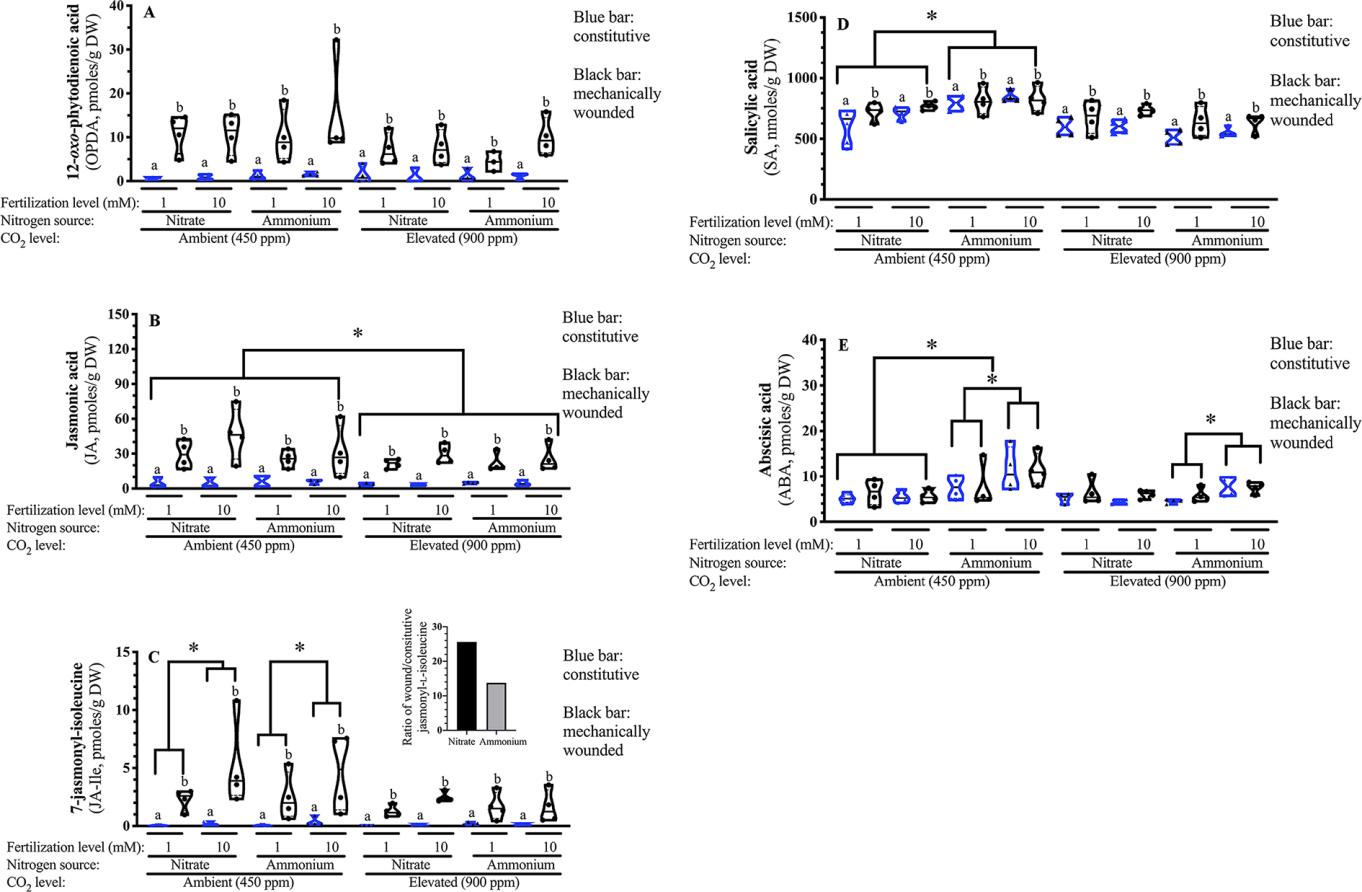
Effect of atmospheric carbon dioxide, nitrogen fertilization and wounding on *Arabidopsis* foliar phytohormone levels. *Arabidopsis thaliana* were grown either at ambient (aCO_2_, 450 ppm) or elevated (eCO_2_, 900 ppm) carbon dioxide and fertilized with either nitrate or ammonium ion (1 or 10 mM). Rosette tissue of a subset of plants was mechanically damaged. Foliar **(A)** 12-*oxo*-phytodienoic acid (OPDA) **(B)** Jasmonic acid (JA) **(C)** 7-Jasmonyl-isoleucine (JA-Ile) **(D)** Salicylic acid (SA) **(E)** Abscisic acid (ABA). Constitutive phytohormone levels are indicated by clear blue boxes. Wound-induced phytohormone levels are indicated by black boxes. The experiment was repeated 4 times and outliers removed by the maximal normed residual test (n = 3–4). Data was transformed as follows: OPDA and JA data were squared and the log_10_ of JA-Ile and ABA data was used. Statistical differences in foliar OPDA, JA and JA-Ile levels were determined by four-factor MANOVA (treatments: nitrogen source, nitrogen level, CO_2_, mechanical damage; p ≤ 0.05) followed by Tukey honestly significant difference *post-hoc* test. Statistical differences in foliar SA and ABA levels was determined by four-factor ANOVA (treatments: nitrogen source, nitrogen level, CO_2_, mechanical damage; p ≤ 0.05) followed by Tukey honestly significant difference *post-hoc* test. Data is represented by violin plots with the median indicated by a solid line and the interquartile by dashed lines. Significant wound-related differences are indicated by alphabetical letters. Other significant differences are indicated by asterisks (*). DW, dry weight.

Given that OPDA, JA and JA-Ile are biosynthetically related, a MANOVA was used to compare treatments on metabolite levels. In response to wounding, a robust increase in all three jasmonates is observed [square root OPDA F_(1,58)_ = 102.21 *p* < 0.001; square root JA F_(1,58)_ = 52.52 *p* < 0.001; log10 JA-Ile F_(1,58)_ = 295.25 *p* < 0.001] (**Figure 6A**).

Lower foliar JA levels are observed in plants grown at eCO_2_ [square root JA F_(1,58)_ = 5.510 *p* = 0.023]; this reflects a 25% reduction in JA levels compared to plants grown at aCO_2_ (**Figure 6B**).

Wound-induced JA-Ile levels vary with nitrogen source; the JA-Ile burst is higher in nitrate-fertilized than ammonium-fertilized plants [log_10_ JA-Ile F_(1,58)_ = 5.71 *p* = 0.021]. Higher levels are observed in well-fertilized plants [log_10_ JA-Ile F_(1,58)_ = 4.91 *p* = 0.032]; however, this increase in JA-Ile levels seen in response to nitrogen fertilization in plants grown at aCO_2_ is attenuated at eCO_2_ [CO_2_ × N level; log_10_ JA-Ile F_(1,58)_ = 4.51 *p* = 0.040]. At ambient CO_2_, JA-Ile levels were approximately double in well fertilized plants; this enhancement was not observed in plants grown at eCO_2_.

Together, these data suggest that at aCO_2_, higher nitrogen fertilization rates result in a stronger jasmonate burst in response to wounding of foliar tissue. Fertilizer effects the magnitude of the 7-jasmonyl-isoleucine burst, which is higher in nitrate-fertilized plants. This strong increase, regardless of the fertilizer source, ammonium or nitrate, is suppressed at eCO_2_ (**Figures 6B**, **C**).

#### Salicylic Acid

SA levels are 20% lower in plants grown under eCO_2_ [F_(1,63)_ = 46.97 *p* < 0.001] (**Figure 6D**). As seen previously (Ogawa et al., 2010), SA levels are induced in response to wounding [F_(1,63)_ = 10.73 *p* = 0.002]. A CO_2_ × nitrogen source interaction is observed (F_(1,63)_ = 17.425 *p* < 0.001); the increased foliar SA levels in response to ammonium ion fertilizer seen in *Arabidopsis* grown at aCO_2_ is not observed at eCO_2_.

#### Abscisic Acid

Foliar ABA levels are affected by nitrogen source and level and atmospheric CO_2_ [Log_10_ nitrogen source F_(1,63)_ = 16.77 *p* < 0.001; nitrogen level F_(1,63)_ = 6.26 *p* = 0.016; CO_2_ F_(1,63)_ = 5.52 *p* = 0.023] (**Figure 6E**). A nitrogen source x nitrogen level interaction is observed where higher ABA levels are observed in response to ammonium fertilization and also enhanced fertilization rate [Log_10_ F_(1,63)_ = 10.01 *p* = 0.003]; this effect is not seen in response to nitrate fertilization. This observation is also particularly striking at aCO_2_; the increase in ABA that reflects nitrogen fertilization is seen at aCO_2_ but not eCO_2_ [Log_10_ CO_2_ x N source F_(1,63)_ = 4.81 *p* = 0.033].

## Discussion

The increase in atmospheric CO_2_ predicted to be reached by the end of the century will have dramatic effects on plant physiology and productivity, particularly of C3 plants. Increase carboxylase activity of Rubisco and decreased photorespiration should enhance plant productivity. However, studies have also found that induced levels of defensive jasmonate phytohormones are suppressed in C3 plants grown at eCO_2_ (Guo et al., 2012; Sun et al., 2013; Vaughan et al., 2014; Li et al., 2016; Paudel. et al., 2016; Lu et al., 2018). Our study has found links between the source of nitrogen, nitrate or ammonium, and cellular reductant levels and redox metabolites. The more oxidized cellular environment of nitrate-fertilized plants translates into a wound-associated increase in the defense-related phytohormone 7-*iso*-jasmonyl-L-isoleucine. However, the fertilizer rate-associate enhancement of the jasmonate burst is damped in plants grown at eCO_2_. Under this condition, ascorbate and salicylic acid levels in wounded leaves possibly contribute to the suppression of the jasmonate burst.

### *Arabidopsis* Foliar Nitrogen Level and Carbon: Nitrogen Ratio Reflect Fertilization Rate

Foliar nitrogen content reflected the fertilizer rate but not the source; higher leaf nitrogen content (%N) and lower C:N ratio is observed in well fertilized plants (10 mM) (**Figures 2B**, **C**). Even though there are reports of decreased total N levels in plants grown at eCO_2_ (Cotrufo et al., 2002; Sun et al., 2002; Yin, 2002; Teng et al., 2006; Bloom et al., 2010), we did not find this; instead fertilizer nitrogen concentration was the major driver effecting foliar percent nitrogen. However, this may reflect a difference in the nitrate concentration that was used in the experiments. In Bloom et al. (2010), nitrate assimilation was inhibited at eCO_2_ when *Arabidopsis* plants were fertilized with relatively low nitrate rates (0.2–1 mM) compared to this study (1–10 mM). Other studies have also found that atmospheric CO_2_ levels have a minimal effect on nitrogen assimilation (Andrews et al., 2019).

### Nitrogen Source Affects *Arabidopsis* Carbon and Nitrogen, Reductant and Redox Metabolite Levels

The leaves of nitrate-fertilized plants have lower NADH levels that may reflect the additional requirements of reductant for nitrate reduction and assimilation as compared to ammonium (**Figure 3A**); these results are in agreement with Podgórska et al. (2015), who observed a decrease in the NAD(P)^+^/NAD(P)H ratio in ammonium-fertilized *Arabidopsis* plants in comparison to nitrate. Compared to ammonium fertilize, the use of nitrate fertilizer by the plant is energetically expensive and requires an additional 8 e^-^/mole nitrogen for nitrate assimilation (Williams et al., 1987; Noctor and Foyer, 1998; Bloom, 2015b). In general, nitrate-fertilized plants have lower CO_2_ assimilation rates, that would translate into lower percent carbon, because of the competition with the CBB cycle for reductant (Scheibe, 2004; Scheibe et al., 2005; Bloom, 2015b). Thus, plants fertilized with nitrate had a lower foliar percent carbon compared to ammonium-fertilized plants (**Figure 3A**).

A number of studies have observed increased mitochondrial respiration and cellular oxidative state in ammonium-fertilized plants, which reflects higher levels of mitochondria-associated reactive oxygen species (Guo et al., 2005; Patterson et al., 2010; Podgórska et al., 2013; Podgórska et al., 2015; Hachiya and Sakakibara, 2017). In ammonium-fertilized plants, excess reducing equivalents must be oxidized by the mitochondria resulting in increased ROS produced by the electron transport chain; It should also be noted that even though under today’s conditions, mitochondrial-generated ROS is significantly less than the ROS produced through photorespiration and photorespiration will be suppressed in C_3_ plants grown under eCO_2_ (Foyer et al., 2011). We found that foliar GSSG, GSH and total glutathione levels are higher in plants fertilized with ammonium than nitrate, but there was no difference in the GSSG/GSH ratio (**Figures 3B**–**E**). Also, total ascorbate levels are lower in ammonium-fertilized plants (**Figure 3F**). Similar to our study, a number of other investigations have also found an increase in total glutathione levels in ammonium-fertilized *Arabidopsis* compared to nitrate-treated plants (Medici et al., 2004; Podgórska et al., 2015); though the ratio of GSSG/GSH was also higher in Medici et al. (2004) study which we did not observe but this study was focused on root tissues. Often, a negative correlation between glutathione and ascorbate exists and, though there is some variability in the literature, lower foliar ascorbate levels in ammonium-fertilized plants have also been reported by others (Kerchev et al., 2011; Podgórska et al., 2015). In fact, decreasing nitrate fertilization is used as a strategy to increase fruit and vegetable vitamin C (ascorbate) content (Stefanelli et al., 2010*)*. Together, these data support the observation that the cellular redox state is more oxidized in nitrate-fertilized plants compared to ammonium (**Figures 3A**–**F**).

### Nitrogen Source Affects *Arabidopsis* Phytohormones in Plants Grown at aCO_2_ (450 ppm)

SA, a key hormone involved in signaling defense pathway against biotrophic pathogens (Boatwright and Pajerowska-Mukhtar, 2013), is also recognized to play an antagonistic role against jasmonate signaling allowing the plant to respond appropriately to environmental stresses (Caarls et al., 2015). SA levels moderate jasmonate levels through phytohormone cross-talk patterns; often a reciprocal, negative correlation between these two hormones is observed (Caarls et al., 2015). Foliar NADH and SA levels were higher in ammonium-fertilized *Arabidopsis* (**Figures 3A** and **6D**). A number of studies have found that increased foliar NADH levels are correlated with higher SA pools and enhance resistance to avirulent biotrophic pathogens as well as a recent study using chemically-induced expression of the ADP-ribose/NADH pyrophosphohydolase AtNUDX7 that also found a positive relationship between foliar NADH and SA levels (Noctor et al., 2011; Pétriacq et al., 2012; Ogawa et al., 2016). Therefore, there may be a relationship between the higher NADH levels in the ammonium-fertilized plants that lead to higher constitutive SA levels observed here (**Figure 3A**); however, it is unclear why this fertilizer-associated effect is attenuated in plants grown at eCO_2_. One possibility is that at eCO_2_, reduced photorespiration may affect the localization of reductant that is linked to SA biosynthesis.

Though best recognized for the key regulatory roles in abiotic stresses, such as cold, drought and salinity (Sah et al., 2016), ABA is also involved in plant responses to mechanical and insect damage (Erb et al., 2012). ABA acts antagonistically to the Erf/ORA59 branch of the jasmonate-dependent signaling pathway that leads to defenses such as defensin (PDF1.2) (Kazan 2018). ABA also attenuates SA-mediated defense responses by stimulating the 26*S*-proteasome-mediated degradation of NPR1 (Ding et al., 2016). In plants grown at aCO_2_, higher ABA levels are also observed in ammonium-fertilized plants and this is more pronounced under a high fertilizer rate (**Figure 6E**); this may reflect the relationship with ascorbate which is lower in ammonium-fertilized plants as a negative correlation is often observed between ascorbate and ABA levels (**Figure 3F**) (Pastori et al., 2003; Kerchev et al., 2011). It is unclear why this pattern is only observed in plants grown at aCO_2_, however, other studies have also found lower ABA levels in plants grown at eCO_2_ (Teng et al., 2006).

### Wounding of *Arabidopsis* Leaves Results in an Increased Oxidative State and Elevated Phytohormone Levels

Dynamic changes in redox metabolites and pyrrolidine nucleotide levels occurred in wounded *Arabidopsis* leaves. Initially, an increase in DHA/Asc occurred within 15 min of wounding that reflected an increase in oxidized ascorbate (DHA) and decrease in reduced ascorbate (Asc) (**Figures 4A**–**C**). In a similar manner, Suza et al. (2010) observed that crushing of *Arabidopsis* leaves with a haemostat increased total ascorbate levels over the initial 6 h period after treatment. In our time course, this is followed by a decrease in NADP^+^/NADPH ratio and an increase in GSSG/GSH and, at 45 min after wounding, an increase in foliar GSSG levels continues (**Figures 4D**–**F**). Together our data indicates an increase in cellular oxidative state in damaged leaves. GSSG accumulation is often linked with increases in SA levels (Mhamdi et al., 2010; Mhamdi et al., 2013); we observed a consistent, slight increase in wound-induced SA levels that may reflect GSSG levels (**Figure 6D**).

Han et al. (2013a) have proposed that glutathione is key for the upregulation of the SA and jasmonate phytohormone pathways and the cross-talk between them. Mechanical dsamage of *Arabidopsis* leaves results in a vigorous increase in jasmonate-related phytohormones (Glauser et al., 2008; Wasternack and Hause, 2013); known as the jasmonate burst. Key representatives of these hormones, that are involved in regulation of plant responses to wounding, are 12-*oxo*-phytodienoic acid (OPDA), jasmonic acid and 7-jasmonyl-isoleucine (JA-Ile). We observed a robust increase in these phytohormones in wounded plants (**Figures 6A**–**C**).

### The Wound-Induced Jasmonate Burst is Attenuated in *Arabidopsis* Grown at eCO_2_ (900 ppm)

Atmospheric CO_2_ affects how *Arabidopsis* responds to mechanical damage. In the hour after damage, foliar NADP levels decline in wounded plants grown at aCO_2_ (**Figure 5A**). As well there is a concomitant increase in NAD levels in wounded *Arabidopsis* grown at eCO_2_ and fertilized with ammonium (**Figure 5B**); therefore, in plants grown at eCO_2_, that will reduce photorespiration, NAD levels in nitrate-fertilized plants is lower than in ammonium-fertilized plants which reflects the increased need for reductant in nitrate assimilation. Reduced and total ascorbate levels increase in the damaged leaves of these plants grown at eCO_2_ (**Figures 5C**, **D**).

The jasmonate burst is the hallmark of the plant defense signaling pathway leading to resistance against chewing herbivores and necrotrophic pathogens (Wasternack and Hause, 2013; Howe et al., 2018). However, this burst is strongly attenuated in wounded plants grown at eCO_2_ (**Figures 6B**, **C**). Early steps in the pathway, *i.e.* OPDA levels, are not affected by fertilizer or CO_2_ levels, suggesting that regulation of this pathway occur after OPDA biosynthesis (**Figure 6A**). In contrast, lower JA levels are observed at eCO_2_, which has also been noted in other studies (Guo et al., 2012; Sun et al., 2013; Vaughan et al., 2014; Li et al., 2016; Lu et al., 2018). As well, the wound-associated increase in the bioactive JA-Ile levels that positively reflects nitrogen fertilizer and rate is not observed in *Arabidopsis* grown at eCO_2_ (**Figure 6C**). It is of interest that the wound-induced increase in JA-Ile is higher in nitrate- than ammonium-fertilized plants which likely reflects the more oxidative state in these plants (**Figure 6C**, inset). Plants grown at aCO_2_ show a more robust JA-Ile increase in well- fertilized plants that is attenuated at eCO_2_, an observation similar to that reported by Paudel et al. (2016) who also noted that the jasmonate burst was attenuated at eCO_2_ and more markedly in well-fertilized plants.

High constitutive SA levels observed in *Arabidopsis* grown at eCO_2_ is proposed to be responsible for the decreased jasmonate levels also observed at eCO_2_ (Casteel et al., 2012a; Zavala et al., 2013; Mhamdi and Noctor, 2016; Gog et al., 2019). Numerous plants species grown at elevated CO_2_ have increased SA levels (Casteel et al., 2012a; Huang et al., 2012; Sun et al., 2013; Mhamdi et al., 2013; Zhang et al., 2015; Li et al., 2019; Gog et al., 2019); though this appears to be plant species- or cultivar-specific and influenced by other environmental factors (Matros et al., 2006; Casteel et al., 2012b; Mhamdi and Noctor, 2016; Noctor and Mhamdi, 2017; Zhou et al., 2019). In fact, in our study, a decrease in SA levels are observed at eCO_2_ which may reflect the photoperiod used in these experiments (12:12 L:D, **Figure 6D**), compared to others conducted under long day conditions (16:8) (Matros et al., 2006; Sun et al., 2013; Zhang et al., 2015; Mhamdi and Noctor, 2016; Gog et al., 2019). *Arabidopsis* plants that have higher SA levels also have increased resistance to hemi-biotrophic and nectrotrophic pathogens, such as *Pseudomonas syringae* DC3000 and *Botrytis cinerea* (Mhamdi and Noctor, 2016); however, a recent study found that resistance of *Arabidopsis* to *P. syringae* pv tomato was reduced in plants grown at eCO2 (Zhou et al., 2019), which also points to the involvement of other factors, such as light or nitrogen, in this network. A wound-induced increase in SA levels was observed as has been seen in previous studies; a transient increase in SA levels 24 h after carborundum-induced mechanical damage of *Arabidopsis* leaves has been previously reported (Ogawa et al., 2010). Given the well-established cross-talk between the SA x jasmonate pathways (Caarls et al., 2015), this may, in part, explain the suppression of the jasmonate burst observed in plants grown at eCO_2_ (**Figure 6C**).

## Conclusion

**Figure 7:**
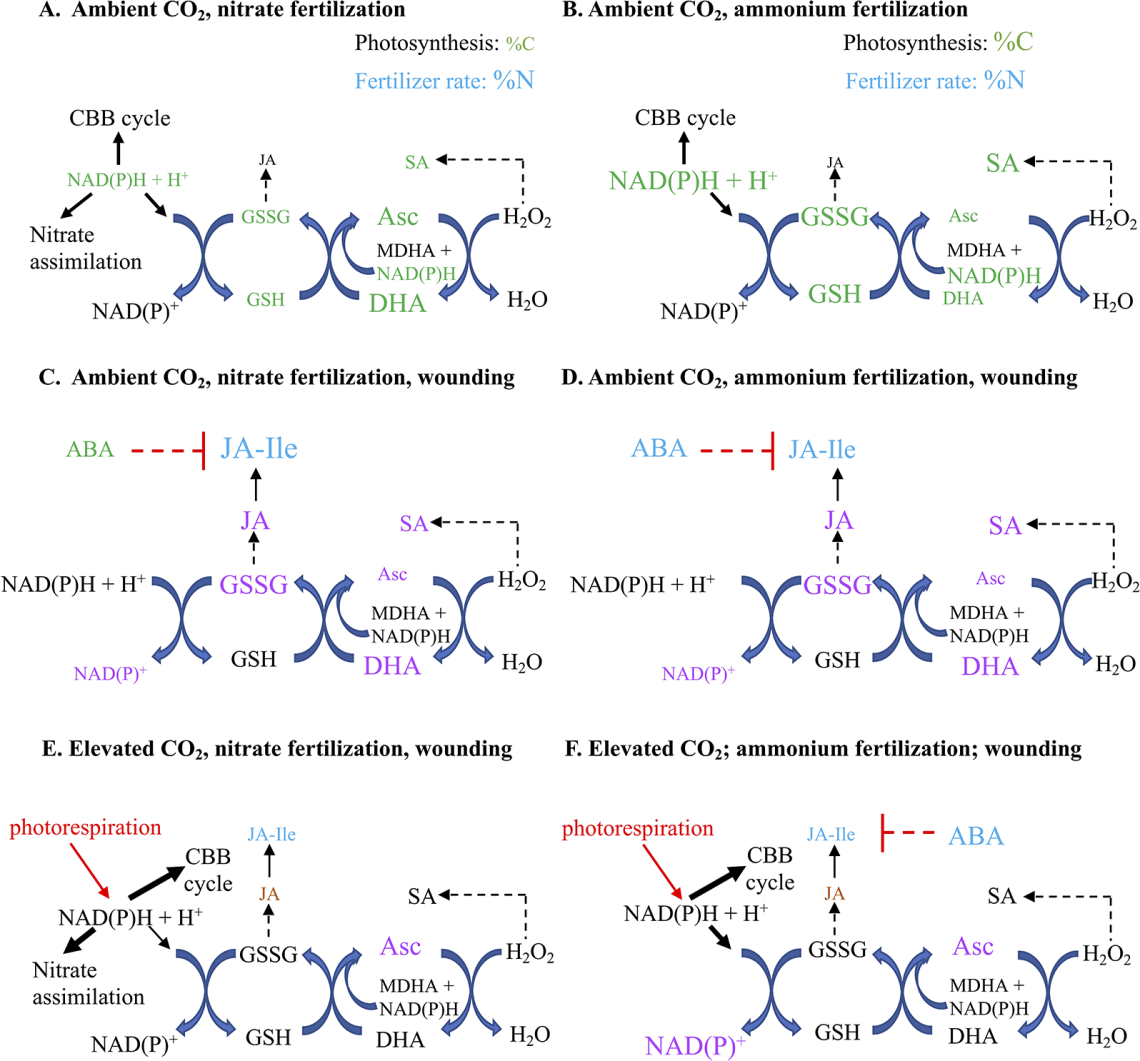
Proposed model of the effect of nitrogen fertilizer on wound-induced foliar defense responses of *Arabidopsis thaliana*, a C3 plant that assimilates nitrate in its leaves, grown under predicted future carbon dioxide levels. Foliar percent carbon (%C) and nitrogen (%N), redox metabolite and pyridine nucleotide levels are represented in *Arabidopsis* fertilized by **(A)** nitrate or **(B)** ammonium. Overall, this reflected a slightly higher oxidative cellular state in plants fertilized by nitrate, which may explain the higher wound-induced JA-Ile levels in **(C)** nitrate compared to **(D)** ammonium fertilized plants (**Figure 6C** inset). At elevated carbon dioxide (eCO_2_) levels, there is a less strong oxidative state in **(E)** nitrate- or **(F)** ammonium-fertilized plants compared to ambient CO_2_ that may, at least partially, account for the suppressed JA-Ile burst in response to wounding. Differences in metabolite levels are indicated by the relative size of their font. The colours represent the treatment effect; green indicates fertilizer type (nitrate or ammonium), blue represents fertilizer rate (1 or 10 mM), purple indicates mechanical damage, red indicates negative interactions and dashed lines indicate putative relationships. ABA, abscisic acid; aCO_2_, ambient carbon dioxide (450 ppm); Asc, reduced form of ascorbate; C, carbon; CBB, Calvin-Benson-Bassham; DHA, dehydroascorbate, oxidized form of ascorbate; eCO_2_, elevated carbon dioxide (900 ppm); GSH, reduced glutathione; GSSG, oxidized glutathione; NAD(P), oxidized form of nicotinamide adenine nucleotide (phosphate); NAD(P)H, reduced form of nicotinamide adenine nucleotide (phosphate); JA, jasmonic acidm JA-Ile: 7-jasmonyl-L-isoleucine; N, nitrogen; OPDA, 12-*oxo*-phytodienoic acid; SA: salicylic acid.

At aCO_2_, overall, a more cellular oxidative state was observed in nitrate-fertilized plants that translates into a higher jasmonate burst in wounded plants (**Figure 7**). In undamaged plants, ammonium fertilization results in higher reductant levels (NADH, **Figure 3A**) and redox buffering capacity (GSSG, GSH, total glutathione; **Figures 3B**–**D**) that likely reflects the requirement for additional reductant in nitrate assimilation. Therefore, in ammonium-fertilized plants, the additional reductant could be used in the CBB cycle that translates into higher carbon fixation (**Figure 2A**). In contrast, total ascorbate levels are higher in nitrate-fertilized plants (**Figure 2F**). Within 15 min after mechanical wounding, an increase in oxidized DHA and concomitant decrease in reduced Asc is observed (**Figures 4A**, **B**). This is followed by an increase in GSSG and decrease in NADP^+^/NADPH levels (**Figures 4E**, **D**). This variance in cellular oxidative state may translate into the noted differences in wound-associated levels of jasmonate and SA phytohormones (**Figures 6A**–**D**). Nitrate-fertilized plants have a more oxidative cellular environment, albeit moderate, compared to ammonium-fertilized plants and this potentially is reflected in the increase in wound-associated JA-Ile levels compared to ammonium-fertilized plants (**Figure 6C**, inset). In addition, the JA-Ile burst is strongest in well-fertilized plants (**Figure 6C**).

At eCO_2_, the decrease in photorespiration is expected to decrease cellular reductant levels which has the potential to effect oxidative signaling in response to wound stress. In response to mechanical damage, the wound-associated decrease in NADP^+^ is only observed in plants grown at aCO_2_ but not eCO_2_ (**Figure 5A**); this may reflect lower NADPH levels due to a decreased photorespiration-associated malate shuttle. In contrast NAD^+^ levels increase in wounded ammonium-fertilized plants grown at eCO_2_, but not aCO_2_ (**Figure 5B**). Ammonium fertilization results in higher foliar NADH levels (**Figure 3A**); the wound-associated increase in NAD^+^ may be more pronounced at eCO_2_ reflecting changes in mitochondrial respiration. As well, there is a wound-associated increase in reduced and total ascorbate levels only observed in plants grown at eCO_2_ (**Figures 5C**, **D**). Therefore, at eCO_2_, in response to wounding, there is less strong oxidative state compared to plants grown at aCO_2_. This translates into a muted JA-Ile burst in response to wounding, particularly in well-fertilized plants grown at eCO_2_, in agreement with previous studies (Paudel et al., 2016) (**Figure 6C**). However, at the fertilization rates used, a difference in wound-induced jasmonates between ammonium- or nitrate-fertilized plants is not observed; therefore, this suppression may be linked to competition for nitrogen assimilation at eCO_2_, but downstream of nitrate reduction and/or foliar ascorbate and SA levels. Wound-induced total ascorbate, which strongly reflects foliar reduced Asc levels, is higher in plants grown at eCO_2_ and a negative correlation between ascorbate and jasmonate signaling pathways has been previously observed (Kerchev et al., 2011). As well, the increase in SA levels induced by wounding may also act to suppress induced jasmonate levels (**Figure 6D**) (Koornneef and Pieterse, 2008; Caarls et al., 2015).

Therefore, in response to damage, dynamic fluctuations in redox metabolites, particular glutathione-related metabolites, indicate an increase in cellular oxidative status in wounded *Arabidopsis* leaves leading to a robust jasmonate burst (**Figures 4A**–**C** and **6A**–**C**). Well-fertilized *Arabidopsis* plants grown at aCO_2_ exhibit stronger JA-Ile increases in damaged leaves, which is heightened in nitrate-fertilized plants, that possibly reflects the cellular oxidative state (**Figures 3A**–**D**, **3F**, **5B**, and **6C**). In *Arabidopsis* grown at eCO_2_, the oxidative state and jasmonate burst is muted in wounded plants (**Figure 6C**). The increase in ascorbate and SA in response to wounding may contribute to the suppression of the JA burst (**Figures 5C** and **6D**).

Plants are sophisticated in their approaches to integrate and respond to everchanging environmental conditions. In the next 50 years, dramatic increases in atmospheric CO_2_ levels are predicted to occur and this will greatly impact plants, particularly those who conduct C_3_ photosynthesis. Understanding how eCO_2_ will impact future plant-interactions, particularly those in response to negative biotic stresses, such as chewing insects or necrotrophic pathogens, is critical to develop sustainable management practices for agricultural crop protection.

## Data Availability Statement

The datasets generated for this study are available on request to the corresponding author.

## Author Contributions

Conceived and designed the experiments: JM and JB. Performed the experiments: JH, LD, KG, and AS. Analyzed the data: JM and JB. Wrote the manuscript: JM, KG, AS, ND, and JB.

## Conflict of Interest

The authors declare that the research was conducted in the absence of any commercial or financial relationships that could be construed as a potential conflict of interest.

## Abbreviations

ABA, abscisic acid; aCO_2_, ambient carbon dioxide (450 ppm); ANOVA, analysis of variance; Asc, reduced form of ascorbate; C, carbon; CBB, Calvin-Benson-Bassham; CO_2_, carbon dioxide; DHA, dehydroascorbate, oxidized form of ascorbate; DW, dry weight; eCO_2_, elevated carbon dioxide (900 ppm); Fd, ferredoxin; FNR, ferredoxin-dependent NADPH reductase; FW, frozen weight; GSH, reduced glutathione; GSSG, oxidized glutathione; ICS1, isochorismate synthase1; NAD, oxidized form of nicotinamide adenine nucleotide; NADH, reduced form of nicotinamide adenine nucleotide; NADP, oxidized form of nicotinamide adenine nucleotide phosphate; NADPH, reduced form of nicotinamide adenine nucleotide phosphate; NH_4_^+^, ammonium ion; NO_3_^-^, nitrate; JA, jasmonic acid; JA-Ile, 7-jasmonyl-L-isoleucine; MANOVA, multivariate analysis of variance; **N**, nitrogen; NPR1, nonexpressor of pathogenesis-related protein1; OPDA, 12-*oxo*-phytodienoic acid; ROS, reactive oxygen species; SA, salicylic acid.
