# Supplementary material for: Fertilizer Rate-Associated Increase in Foliar Jasmonate Burst Observed in Wounded Arabidopsis thaliana Leaves is Attenuated at eCO2
Source: Front Plant Sci. 2020 Jan 16;10:1636. doi: 10.3389/fpls.2019.01636 (PMC6977439; doi:10.3389/fpls.2019.01636)
Supplement: Supplementary file 1 [file DataSheet_1.docx]

**Supplemental Table 1.** Osmolarity and osmotic pressure of the different fertilizer solutions used in experimental treatments and nutrient controls.

| Nitrogen source | Solution osmolality and osmotic pressure | Nitrogen concentration | | | |
| --- | --- | --- | --- | --- | --- |
|  |  | Experimental | | Nutrient control | |
|  |  | 1 mM | 10 mM | 1 mM | K_2_SO_4_ |
| KNO_3_ | Osmolality (mOsmol/L) | 10.84 | 28.84 | 47.64 | 9.2 mM |
|  | Osmotic pressure (atm) | 0.26 | 0.69 | 1.14 |  |
| (NH_4_)_2_SO_4_ | Osmolality (mOsmol/L) | 11.84 | 38.84 | 47.84 | 9 mM |
|  | Osmotic pressure (atm) | 0.28 | 0.93 | 1.15 |  |

**Supplemental Table 2.** Transitions or specific pair of m/z values associated to the precursors and fragment ions of the analytes measured by high performance liquid chromatography-mass spectrometry (LC-MS).

| **Analyte** | **Q1 [m/z] → Q3 [m/z]^a^** | **Collision Energy [V]** | **Standard** |
| --- | --- | --- | --- |
| ABA | (-) 263.13 → 153.00 | 9 | D6-ABA |
| JA | (-) 209.12 → 59.00 | 12 | D6-JA |
| JA-Ile | (-) 322.20 → 130.00 | 19 | D6-JA-Ile |
| SA | (-) 137.02 → 93.00 | 15 | D4-SA |
| D6-ABA | (-) 269.17 → 159.00 | 10 |  |
| D6-JA | (-) 215.15 → 59.00 | 10 |  |
| D6-JA-Ile | (-) 328.24 → 130.00 | 19 |  |
| D4-SA | (-) 141.05 → 97.00 | 15 |  |
| OPDA | (-) 291.00 → 165.00 | 18 | D6-JA-Ile |

a: Resolution: Q1: 0.7, Q3:22

b: Analyzed as the sum of all three transitions

**Supplemental Table 3. Counterion controls**. Fertilizers contained either ammonium ion ((NH_4_)_2_SO_4_) or nitrate (KNO_3_). Controls were conducted to ensure that counterions (SO_4_^-^ or K^+^) did not affect experimental results. Log_10_ transformations were conducted for ratios. Treatment and counterion controls were compared by Student’s t-test. A Bonferroni correction was used, therefore, significant differences require *p* ≤

0.0002 (Armstrong, 2014).

|  |  |  |  | Degrees of freedom | Significance (equal variances assumed) |
| --- | --- | --- | --- | --- | --- |
| **Carbon (C)** |  |  |  |  |  |
| aCO_2_ | NH_4_^+^ |  |  | Df = 4 | *p* = 0.660 |
| aCO_2_ | NO_3_^-^ |  |  | Df = 4 | *p* = 0.289 |
| eCO_2_ | NH_4_^+^ |  |  | Df = 5 | *p* = 0.935 |
| eCO_2_ | NO_3_^-^ |  |  | Df = 4 | *p* = 0.452 |
|  |  |  |  |  |  |
| **Nitrogen (N)** |  |  |  |  |  |
| aCO_2_ | NH_4_^+^ |  |  | Df = 4 | *p* = 0.422 |
| aCO_2_ | NO_3_^-^ |  |  | Df = 4 | *p* = 0.653 |
| eCO_2_ | NH_4_^+^ |  |  | Df = 5 | *p* = 0.513 |
| eCO_2_ | NO_3_^-^ |  |  | Df = 4 | *p* = 0.517 |
|  |  |  |  |  |  |
| **Log_10_ C:N** |  |  |  |  |  |
| aCO_2_ | NH_4_^+^ |  |  | Df = 4 | *p* = 0.221 |
| aCO_2_ | NO_3_^-^ |  |  | Df = 4 | *p* = 0.894 |
| eCO_2_ | NH_4_^+^ |  |  | Df = 5 | *p* = 0.289 |
| eCO_2_ | NO_3_^-^ |  |  | Df = 4 | *p* = 0.475 |
|  |  |  |  |  |  |
| **NAD^+^** |  |  |  |  |  |
| aCO_2_ | NH_4_^+^ | 15 min | unwounded | Df = 7 | *p* = 0.621 |
| aCO_2_ | NO_3_^-^ | 15 min | unwounded | Df = 6 | *p* = 0.762 |
| aCO_2_ | NH_4_^+^ | 15 min | wounded | Df = 7 | *p* = 0.242 |
| aCO_2_ | NO_3_^-^ | 15 min | wounded | Df = 8 | *p* = 0.382 |
| aCO_2_ | NH_4_^+^ | 30 min | unwounded | Df = 6 | *p* = 0.293 |
| aCO_2_ | NO_3_^-^ | 30 min | unwounded | Df = 6 | *p* = 0.955 |
| aCO_2_ | NH_4_^+^ | 30 min | wounded | Df = 5 | *p* = 0.028 |
| aCO_2_ | NO_3_^-^ | 30 min | wounded | Df = 6 | *p* = 0.716 |
| aCO_2_ | NH_4_^+^ | 45 min | unwounded | Df = 6 | *p* = 0.566 |
| aCO_2_ | NO_3_^-^ | 45 min | unwounded | Df = 4 | *p* = 0.099 |
| aCO_2_ | NH_4_^+^ | 45 min | wounded | Df = 5 | *p* = 0.910 |
| aCO_2_ | NO_3_^-^ | 45 min | wounded | Df = 6 | *p* = 0.177 |
| eCO_2_ | NH_4_^+^ | 15 min | unwounded | Df = 6 | *p* = 0.694 |
| eCO_2_ | NO_3_^-^ | 15 min | unwounded | Df = 7 | *p* = 0.379 |
| eCO_2_ | NH_4_^+^ | 15 min | wounded | Df = 7 | *p* = 0.495 |
| eCO_2_ | NO_3_^-^ | 15 min | wounded | Df = 8 | *p* = 0.272 |
| eCO_2_ | NH_4_^+^ | 30 min | unwounded | Df = 6 | *p* = 0.719 |
| eCO_2_ | NO_3_^-^ | 30 min | unwounded | Df = 6 | *p* = 0.640 |
| eCO_2_ | NH_4_^+^ | 30 min | wounded | Df = 6 | *p* = 0.998 |
| eCO_2_ | NO_3_^-^ | 30 min | wounded | Df = 6 | *p* = 0.064 |
| eCO_2_ | NH_4_^+^ | 45 min | unwounded | Df = 6 | *p* = 0.104 |
| eCO_2_ | NO_3_^-^ | 45 min | unwounded | Df = 6 | *p* = 0.405 |
| eCO_2_ | NH_4_^+^ | 45 min | wounded | Df = 5 | *p* = 0.350 |
| eCO_2_ | NO_3_^-^ | 45 min | wounded | Df = 6 | *p* = 0.373 |
|  |  |  |  |  |  |
| **NADH** |  |  |  |  |  |
| aCO_2_ | NH_4_^+^ | 15 min | unwounded | Df = 7 | *p* = 0.289 |
| aCO_2_ | NO_3_^-^ | 15 min | unwounded | Df = 6 | *p* = 0.067 |
| aCO_2_ | NH_4_^+^ | 15 min | wounded | Df = 7 | *p* = 0.974 |
| aCO_2_ | NO_3_^-^ | 15 min | wounded | Df = 6 | *p* = 0.264 |
| aCO_2_ | NH_4_^+^ | 30 min | unwounded | Df = 6 | *p* = 0.055 |
| aCO_2_ | NO_3_^-^ | 30 min | unwounded | Df = 6 | *p* = 0.770 |
| aCO_2_ | NH_4_^+^ | 30 min | wounded | Df = 6 | *p* = 0.624 |
| aCO_2_ | NO_3_^-^ | 30 min | wounded | Df = 6 | *p* = 0.240 |
| aCO_2_ | NH_4_^+^ | 45 min | unwounded | Df = 6 | *p* = 0.599 |
| aCO_2_ | NO_3_^-^ | 45 min | unwounded | Df = 4 | *p* = 0.030 |
| aCO_2_ | NH_4_^+^ | 45 min | wounded | Df = 5 | *p* = 0.172 |
| aCO_2_ | NO_3_^-^ | 45 min | wounded | Df = 6 | *p* = 0.984 |
| eCO_2_ | NH_4_^+^ | 15 min | unwounded | Df = 6 | *p* = 0.492 |
| eCO_2_ | NO_3_^-^ | 15 min | unwounded | Df = 7 | *p* = 0.780 |
| eCO_2_ | NH_4_^+^ | 15 min | wounded | Df = 7 | *p* = 0.866 |
| eCO_2_ | NO_3_^-^ | 15 min | wounded | Df = 8 | *p* = 0.391 |
| eCO_2_ | NH_4_^+^ | 30 min | unwounded | Df = 6 | *p* = 0.352 |
| eCO_2_ | NO_3_^-^ | 30 min | unwounded | Df = 6 | *p* = 0.663 |
| eCO_2_ | NH_4_^+^ | 30 min | wounded | Df = 6 | *p* = 0.094 |
| eCO_2_ | NO_3_^-^ | 30 min | wounded | Df = 7 | *p* = 0.782 |
| eCO_2_ | NH_4_^+^ | 45 min | unwounded | Df = 6 | *p* = 0.948 |
| eCO_2_ | NO_3_^-^ | 45 min | unwounded | Df = 6 | *p* = 0.502 |
| eCO_2_ | NH_4_^+^ | 45 min | wounded | Df = 5 | *p* = 0.259 |
| eCO_2_ | NO_3_^-^ | 45 min | wounded | Df = 6 | *p* = 0.122 |
|  |  |  |  |  |  |
| **Log NAD^+^/NADH** |  |  |  |  |  |
| aCO_2_ | NH_4_^+^ | 15 min | unwounded | Df = 7 | *p* = 0.416 |
| aCO_2_ | NO_3_^-^ | 15 min | unwounded | Df = 6 | *p* = 0.279 |
| aCO_2_ | NH_4_^+^ | 15 min | wounded | Df = 7 | *p* = 0.335 |
| aCO_2_ | NO_3_^-^ | 15 min | wounded | Df = 6 | *p* = 0.788 |
| aCO_2_ | NH_4_^+^ | 30 min | unwounded | Df = 6 | *p* = 0.041 |
| aCO_2_ | NO_3_^-^ | 30 min | unwounded | Df = 6 | *p* = 0.929 |
| aCO_2_ | NH_4_^+^ | 30 min | wounded | Df = 5 | *p* = 0.548 |
| aCO_2_ | NO_3_^-^ | 30 min | wounded | Df = 6 | *p* = 0.468 |
| aCO_2_ | NH_4_^+^ | 45 min | unwounded | Df = 6 | *p* = 0.375 |
| aCO_2_ | NO_3_^-^ | 45 min | unwounded | Df = 4 | *p* = 0.755 |
| aCO_2_ | NH_4_^+^ | 45 min | wounded | Df = 5 | *p* = 0.050 |
| aCO_2_ | NO_3_^-^ | 45 min | wounded | Df = 6 | *p* = 0.243 |
| eCO_2_ | NH_4_^+^ | 15 min | unwounded | Df = 6 | *p* = 0.388 |
| eCO_2_ | NO_3_^-^ | 15 min | unwounded | Df = 7 | *p* = 0.478 |
| eCO_2_ | NH_4_^+^ | 15 min | wounded | Df = 7 | *p* = 0.686 |
| eCO_2_ | NO_3_^-^ | 15 min | wounded | Df = 8 | *p* = 0.091 |
| eCO_2_ | NH_4_^+^ | 30 min | unwounded | Df = 6 | *p* = 0.793 |
| eCO_2_ | NO_3_^-^ | 30 min | unwounded | Df = 6 | *p* = 0.529 |
| eCO_2_ | NH_4_^+^ | 30 min | wounded | Df = 6 | *p* = 0.113 |
| eCO_2_ | NO_3_^-^ | 30 min | wounded | Df = 6 | *p* = 0.201 |
| eCO_2_ | NH_4_^+^ | 45 min | unwounded | Df = 6 | *p* = 0.536 |
| eCO_2_ | NO_3_^-^ | 45 min | unwounded | Df = 6 | *p* = 0.985 |
| eCO_2_ | NH_4_^+^ | 45 min | wounded | Df = 5 | *p* = 0.074 |
| eCO_2_ | NO_3_^-^ | 45 min | wounded | Df = 6 | *p* = 0.094 |
|  |  |  |  |  |  |
| **NADP^+^** |  |  |  |  |  |
| aCO_2_ | NH_4_^+^ | 15 min | unwounded | Df = 7 | *p* = 0.683 |
| aCO_2_ | NO_3_^-^ | 15 min | unwounded | Df = 4 | *p* = 0.654 |
| aCO_2_ | NH_4_^+^ | 15 min | wounded | Df = 7 | *p* = 0.447 |
| aCO_2_ | NO_3_^-^ | 15 min | wounded | Df = 8 | *p* = 0.845 |
| aCO_2_ | NH_4_^+^ | 30 min | unwounded | Df = 6 | *p* = 0.602 |
| aCO_2_ | NO_3_^-^ | 30 min | unwounded | Df = 5 | *p* = 0.956 |
| aCO_2_ | NH_4_^+^ | 30 min | wounded | Df = 6 | *p* = 0.324 |
| aCO_2_ | NO_3_^-^ | 30 min | wounded | Df = 6 | *p* = 0.262 |
| aCO_2_ | NH_4_^+^ | 45 min | unwounded | Df = 6 | *p* = 0.162 |
| aCO_2_ | NO_3_^-^ | 45 min | unwounded | Df = 4 | *p* = 0.110 |
| aCO_2_ | NH_4_^+^ | 45 min | wounded | Df = 6 | *p* = 0.868 |
| aCO_2_ | NO_3_^-^ | 45 min | wounded | Df = 6 | *p* = 0.099 |
| eCO_2_ | NH_4_^+^ | 15 min | unwounded | Df = 6 | *p* = 0.855 |
| eCO_2_ | NO_3_^-^ | 15 min | unwounded | Df = 7 | *p* = 0.970 |
| eCO_2_ | NH_4_^+^ | 15 min | wounded | Df = 7 | *p* = 0.919 |
| eCO_2_ | NO_3_^-^ | 15 min | wounded | Df = 8 | *p* = 0.683 |
| eCO_2_ | NH_4_^+^ | 30 min | unwounded | Df = 6 | *p* = 0.856 |
| eCO_2_ | NO_3_^-^ | 30 min | unwounded | Df = 6 | *p* = 0.422 |
| eCO_2_ | NH_4_^+^ | 30 min | wounded | Df = 6 | *p* = 0.983 |
| eCO_2_ | NO_3_^-^ | 30 min | wounded | Df = 5 | *p* = 0.585 |
| eCO_2_ | NH_4_^+^ | 45 min | unwounded | Df = 6 | *p* = 0.769 |
| eCO_2_ | NO_3_^-^ | 45 min | unwounded | Df = 6 | *p* = 0.637 |
| eCO_2_ | NH_4_^+^ | 45 min | wounded | Df = 6 | *p* = 0.739 |
| eCO_2_ | NO_3_^-^ | 45 min | wounded | Df = 5 | *p* = 0.831 |
|  |  |  |  |  |  |
| **NADPH** |  |  |  |  |  |
| aCO_2_ | NH_4_^+^ | 15 min | unwounded | Df = 7 | *p* = 0.242 |
| aCO_2_ | NO_3_^-^ | 15 min | unwounded | Df = 4 | *p* = 0.024 |
| aCO_2_ | NH_4_^+^ | 15 min | wounded | Df = 7 | *p* = 0.049 |
| aCO_2_ | NO_3_^-^ | 15 min | wounded | Df = 6 | *p* = 0.362 |
| aCO_2_ | NH_4_^+^ | 30 min | unwounded | Df = 6 | *p* = 0.044 |
| aCO_2_ | NO_3_^-^ | 30 min | unwounded | Df = 6 | *p* = 0.407 |
| aCO_2_ | NH_4_^+^ | 30 min | wounded | Df = 6 | *p* = 0.213 |
| aCO_2_ | NO_3_^-^ | 30 min | wounded | Df = 5 | *p* = 0.986 |
| aCO_2_ | NH_4_^+^ | 45 min | unwounded | Df = 6 | *p* = 0.908 |
| aCO_2_ | NO_3_^-^ | 45 min | unwounded | Df = 4 | *p* = 0.487 |
| aCO_2_ | NH_4_^+^ | 45 min | wounded | Df = 6 | *p* = 0.979 |
| aCO_2_ | NO_3_^-^ | 45 min | wounded | Df = 6 | *p* = 0.911 |
| eCO_2_ | NH_4_^+^ | 15 min | unwounded | Df = 6 | *p* = 0.225 |
| eCO_2_ | NO_3_^-^ | 15 min | unwounded | Df = 7 | *p* = 0.442 |
| eCO_2_ | NH_4_^+^ | 15 min | wounded | Df = 7 | *p* = 0.083 |
| eCO_2_ | NO_3_^-^ | 15 min | wounded | Df = 8 | *p* = 0.329 |
| eCO_2_ | NH_4_^+^ | 30 min | unwounded | Df = 6 | *p* = 0.558 |
| eCO_2_ | NO_3_^-^ | 30 min | unwounded | Df = 6 | *p* = 0.889 |
| eCO_2_ | NH_4_^+^ | 30 min | wounded | Df = 6 | *p* = 0.303 |
| eCO_2_ | NO_3_^-^ | 30 min | wounded | Df = 7 | *p* = 0.658 |
| eCO_2_ | NH_4_^+^ | 45 min | unwounded | Df = 6 | *p* = 0.339 |
| eCO_2_ | NO_3_^-^ | 45 min | unwounded | Df = 6 | *p* = 0.604 |
| eCO_2_ | NH_4_^+^ | 45 min | wounded | Df = 6 | *p* = 0.777 |
| eCO_2_ | NO_3_^-^ | 45 min | wounded | Df = 6 | *p* = 0.792 |
|  |  |  |  |  |  |
| **Log NADP^+^/NADPH** |  |  |  |  |  |
| aCO_2_ | NH_4_^+^ | 15 min | unwounded | Df = 7 | *p* = 0.791 |
| aCO_2_ | NO_3_^-^ | 15 min | unwounded | Df = 4 | *p* = 0.688 |
| aCO_2_ | NH_4_^+^ | 15 min | wounded | Df = 7 | *p* = 0.964 |
| aCO_2_ | NO_3_^-^ | 15 min | wounded | Df = 6 | *p* = 0.741 |
| aCO_2_ | NH_4_^+^ | 30 min | unwounded | Df = 6 | *p* = 0.025 |
| aCO_2_ | NO_3_^-^ | 30 min | unwounded | Df = 5 | *p* = 0.952 |
| aCO_2_ | NH_4_^+^ | 30 min | wounded | Df = 6 | *p* = 0.063 |
| aCO_2_ | NO_3_^-^ | 30 min | wounded | Df = 6 | *p* = 0.998 |
| aCO_2_ | NH_4_^+^ | 45 min | unwounded | Df = 6 | *p* = 0.369 |
| aCO_2_ | NO_3_^-^ | 45 min | unwounded | Df = 4 | *p* = 0.125 |
| aCO_2_ | NH_4_^+^ | 45 min | wounded | Df = 6 | *p* = 0.861 |
| aCO_2_ | NO_3_^-^ | 45 min | wounded | Df = 6 | *p* = 0.421 |
| eCO_2_ | NH_4_^+^ | 15 min | unwounded | Df = 6 | *p* = 0.811 |
| eCO_2_ | NO_3_^-^ | 15 min | unwounded | Df = 7 | *p* = 0.520 |
| eCO_2_ | NH_4_^+^ | 15 min | wounded | Df = 7 | *p* = 0.341 |
| eCO_2_ | NO_3_^-^ | 15 min | wounded | Df = 8 | *p* = 0.846 |
| eCO_2_ | NH_4_^+^ | 30 min | unwounded | Df = 6 | *p* = 0.560 |
| eCO_2_ | NO_3_^-^ | 30 min | unwounded | Df = 6 | *p* = 0.624 |
| eCO_2_ | NH_4_^+^ | 30 min | wounded | Df = 6 | *p* = 0.836 |
| eCO_2_ | NO_3_^-^ | 30 min | wounded | Df = 5 | *p* = 0.775 |
| eCO_2_ | NH_4_^+^ | 45 min | unwounded | Df = 6 | *p* = 0.366 |
| eCO_2_ | NO_3_^-^ | 45 min | unwounded | Df = 6 | *p* = 0.367 |
| eCO_2_ | NH_4_^+^ | 45 min | wounded | Df = 6 | *p* = 0.554 |
| eCO_2_ | NO_3_^-^ | 45 min | wounded | Df = 5 | *p* = 0.998 |
|  |  |  |  |  |  |
| **GSH** |  |  |  |  |  |
| aCO_2_ | NH_4_^+^ | 15 min | unwounded | Df = 7 | *p* = 0.195 |
| aCO_2_ | NO_3_^-^ | 15 min | unwounded | Df = 6 | *p* = 0.305 |
| aCO_2_ | NH_4_^+^ | 15 min | wounded | Df = 6 | *p* = 1.000 |
| aCO_2_ | NO_3_^-^ | 15 min | wounded | Df = 6 | *p* = 0.370 |
| aCO_2_ | NH_4_^+^ | 30 min | unwounded | Df = 8 | *p* = 0.649 |
| aCO_2_ | NO_3_^-^ | 30 min | unwounded | Df = 7 | *p* = 0.572 |
| aCO_2_ | NH_4_^+^ | 30 min | wounded | Df = 6 | *p* = 0.487 |
| aCO_2_ | NO_3_^-^ | 30 min | wounded | Df = 6 | *p* = 0.236 |
| aCO_2_ | NH_4_^+^ | 45 min | unwounded | Df = 6 | *p* = 0.251 |
| aCO_2_ | NO_3_^-^ | 45 min | unwounded | Df = 5 | *p* = 0.544 |
| aCO_2_ | NH_4_^+^ | 45 min | wounded | Df = 6 | *p* = 0.646 |
| aCO_2_ | NO_3_^-^ | 45 min | wounded | Df = 6 | *p* = 0.080 |
| eCO_2_ | NH_4_^+^ | 15 min | unwounded | Df = 4 | *p* = 0.615 |
| eCO_2_ | NO_3_^-^ | 15 min | unwounded | Df = 6 | *p* = 0.621 |
| eCO_2_ | NH_4_^+^ | 15 min | wounded | Df = 7 | *p* = 0.280 |
| eCO_2_ | NO_3_^-^ | 15 min | wounded | Df = 7 | *p* = 0.415 |
| eCO_2_ | NH_4_^+^ | 30 min | unwounded | Df = 6 | *p* = 0.398 |
| eCO_2_ | NO_3_^-^ | 30 min | unwounded | Df = 7 | *p* = 0.397 |
| eCO_2_ | NH_4_^+^ | 30 min | wounded | Df = 6 | *p* = 0.803 |
| eCO_2_ | NO_3_^-^ | 30 min | wounded | Df = 4 | *p* = 0.831 |
| eCO_2_ | NH_4_^+^ | 45 min | unwounded | Df = 4 | *p* = 0.099 |
| eCO_2_ | NO_3_^-^ | 45 min | unwounded | Df = 7 | *p* = 0.288 |
| eCO_2_ | NH_4_^+^ | 45 min | wounded | Df = 6 | *p* = 0.297 |
| eCO_2_ | NO_3_^-^ | 45 min | wounded | Df = 6 | *p* = 0.949 |
|  |  |  |  |  |  |
| **GSSG** |  |  |  |  |  |
| aCO_2_ | NH_4_^+^ | 15 min | unwounded | Df = 7 | *p* = 0.076 |
| aCO_2_ | NO_3_^-^ | 15 min | unwounded | Df = 6 | *p* = 0.852 |
| aCO_2_ | NH_4_^+^ | 15 min | wounded | Df = 6 | *p* = 0.146 |
| aCO_2_ | NO_3_^-^ | 15 min | wounded | Df = 6 | *p* = 0.347 |
| aCO_2_ | NH_4_^+^ | 30 min | unwounded | Df = 8 | *p* = 0.199 |
| aCO_2_ | NO_3_^-^ | 30 min | unwounded | Df = 7 | *p* = 0.158 |
| aCO_2_ | NH_4_^+^ | 30 min | wounded | Df = 6 | *p* = 0.897 |
| aCO_2_ | NO_3_^-^ | 30 min | wounded | Df = 6 | *p* = 0.387 |
| aCO_2_ | NH_4_^+^ | 45 min | unwounded | Df = 6 | *p* = 0.721 |
| aCO_2_ | NO_3_^-^ | 45 min | unwounded | Df = 5 | *p* = 0.475 |
| aCO_2_ | NH_4_^+^ | 45 min | wounded | Df = 6 | *p* = 0.600 |
| aCO_2_ | NO_3_^-^ | 45 min | wounded | Df = 6 | *p* = 0.642 |
| eCO_2_ | NH_4_^+^ | 15 min | unwounded | Df = 4 | *p* = 0.014 |
| eCO_2_ | NO_3_^-^ | 15 min | unwounded | Df = 6 | *p* = 0.884 |
| eCO_2_ | NH_4_^+^ | 15 min | wounded | Df = 7 | *p* = 0.919 |
| eCO_2_ | NO_3_^-^ | 15 min | wounded | Df = 7 | *p* = 0.772 |
| eCO_2_ | NH_4_^+^ | 30 min | unwounded | Df = 5 | *p* = 0.174 |
| eCO_2_ | NO_3_^-^ | 30 min | unwounded | Df = 6 | *p* = 0.219 |
| eCO_2_ | NH_4_^+^ | 30 min | wounded | Df = 6 | *p* = 0.794 |
| eCO_2_ | NO_3_^-^ | 30 min | wounded | Df = 4 | *p* = 0.087 |
| eCO_2_ | NH_4_^+^ | 45 min | unwounded | Df = 4 | *p* = 0.544 |
| eCO_2_ | NO_3_^-^ | 45 min | unwounded | Df = 7 | *p* = 0.409 |
| eCO_2_ | NH_4_^+^ | 45 min | wounded | Df = 6 | *p* = 0.900 |
| eCO_2_ | NO_3_^-^ | 45 min | wounded | Df = 6 | *p* = 0.338 |
|  |  |  |  |  |  |
| **Total glutathione** |  |  |  |  |  |
| aCO_2_ | NH_4_^+^ | 15 min | unwounded | Df = 7 | *p* = 0.179 |
| aCO_2_ | NO_3_^-^ | 15 min | unwounded | Df = 6 | *p* = 0.312 |
| aCO_2_ | NH_4_^+^ | 15 min | wounded | Df = 6 | *p* = 0.897 |
| aCO_2_ | NO_3_^-^ | 15 min | wounded | Df = 6 | *p* = 0.320 |
| aCO_2_ | NH_4_^+^ | 30 min | unwounded | Df = 8 | *p* = 0.474 |
| aCO_2_ | NO_3_^-^ | 30 min | unwounded | Df = 7 | *p* = 0.503 |
| aCO_2_ | NH_4_^+^ | 30 min | wounded | Df = 6 | *p* = 0.520 |
| aCO_2_ | NO_3_^-^ | 30 min | wounded | Df = 6 | *p* = 0.309 |
| aCO_2_ | NH_4_^+^ | 45 min | unwounded | Df = 6 | *p* = 0.285 |
| aCO_2_ | NO_3_^-^ | 45 min | unwounded | Df = 5 | *p* = 0.491 |
| aCO_2_ | NH_4_^+^ | 45 min | wounded | Df = 6 | *p* = 0.615 |
| aCO_2_ | NO_3_^-^ | 45 min | wounded | Df = 6 | *p* = 0.122 |
| eCO_2_ | NH_4_^+^ | 15 min | unwounded | Df = 4 | *p* = 0.482 |
| eCO_2_ | NO_3_^-^ | 15 min | unwounded | Df = 6 | *p* = 0.626 |
| eCO_2_ | NH_4_^+^ | 15 min | wounded | Df = 7 | *p* = 0.298 |
| eCO_2_ | NO_3_^-^ | 15 min | wounded | Df = 7 | *p* = 0.440 |
| eCO_2_ | NH_4_^+^ | 30 min | unwounded | Df = 5 | *p* = 0.139 |
| eCO_2_ | NO_3_^-^ | 30 min | unwounded | Df = 7 | *p* = 0.518 |
| eCO_2_ | NH_4_^+^ | 30 min | wounded | Df = 6 | *p* = 0.976 |
| eCO_2_ | NO_3_^-^ | 30 min | wounded | Df = 4 | *p* = 0.945 |
| eCO_2_ | NH_4_^+^ | 45 min | unwounded | Df = 4 | *p* = 0.116 |
| eCO_2_ | NO_3_^-^ | 45 min | unwounded | Df = 7 | *p* = 0.274 |
| eCO_2_ | NH_4_^+^ | 45 min | wounded | Df = 6 | *p* = 0.302 |
| eCO_2_ | NO_3_^-^ | 45 min | wounded | Df = 6 | *p* = 0.896 |
|  |  |  |  |  |  |
| **Log GSSG/GSH** |  |  |  |  |  |
| aCO_2_ | NH_4_^+^ | 15 min | unwounded | Df = 7 | *p* = 0.644 |
| aCO_2_ | NO_3_^-^ | 15 min | unwounded | Df = 4 | *p* = 0.696 |
| aCO_2_ | NH_4_^+^ | 15 min | wounded | Df = 6 | *p* = 0.325 |
| aCO_2_ | NO_3_^-^ | 15 min | wounded | Df = 6 | *p* = 0.914 |
| aCO_2_ | NH_4_^+^ | 30 min | unwounded | Df = 8 | *p* = 0.092 |
| aCO_2_ | NO_3_^-^ | 30 min | unwounded | Df = 7 | *p* = 0.421 |
| aCO_2_ | NH_4_^+^ | 30 min | wounded | Df = 6 | *p* = 0.535 |
| aCO_2_ | NO_3_^-^ | 30 min | wounded | Df = 6 | *p* = 0.170 |
| aCO_2_ | NH_4_^+^ | 45 min | unwounded | Df = 6 | *p* = 0.700 |
| aCO_2_ | NO_3_^-^ | 45 min | unwounded | Df = 5 | *p* = 0.872 |
| aCO_2_ | NH_4_^+^ | 45 min | wounded | Df = 6 | *p* = 0.805 |
| aCO_2_ | NO_3_^-^ | 45 min | wounded | Df = 6 | *p* = 0.624 |
| eCO_2_ | NH_4_^+^ | 15 min | unwounded | Df = 4 | *p* = 0.072 |
| eCO_2_ | NO_3_^-^ | 15 min | unwounded | Df = 6 | *p* = 0.636 |
| eCO_2_ | NH_4_^+^ | 15 min | wounded | Df = 7 | *p* = 0.729 |
| eCO_2_ | NO_3_^-^ | 15 min | wounded | Df = 7 | *p* = 0.185 |
| eCO_2_ | NH_4_^+^ | 30 min | unwounded | Df = 5 | *p* = 0.094 |
| eCO_2_ | NO_3_^-^ | 30 min | unwounded | Df = 6 | *p* = 0.274 |
| eCO_2_ | NH_4_^+^ | 30 min | wounded | Df = 6 | *p* = 0.534 |
| eCO_2_ | NO_3_^-^ | 30 min | wounded | Df = 4 | *p* = 0.133 |
| eCO_2_ | NH_4_^+^ | 45 min | unwounded | Df = 4 | *p* = 0.810 |
| eCO_2_ | NO_3_^-^ | 45 min | unwounded | Df = 7 | *p* = 0.689 |
| eCO_2_ | NH_4_^+^ | 45 min | wounded | Df = 6 | *p* = 0.549 |
| eCO_2_ | NO_3_^-^ | 45 min | wounded | Df = 6 | *p* = 0.474 |
|  |  |  |  |  |  |
| **Asc (reduced)** |  |  |  |  |  |
| aCO_2_ | NH_4_^+^ | 15 min | unwounded | Df = 6 | *p* = 0.489 |
| aCO_2_ | NO_3_^-^ | 15 min | unwounded | Df = 6 | *p* = 0.155 |
| aCO_2_ | NH_4_^+^ | 15 min | wounded | Df = 5 | *p* = 0.471 |
| aCO_2_ | NO_3_^-^ | 15 min | wounded | Df = 6 | *p* = 0.820 |
| aCO_2_ | NH_4_^+^ | 30 min | unwounded | Df = 7 | *p* = 0.090 |
| aCO_2_ | NO_3_^-^ | 30 min | unwounded | Df = 6 | *p* = 0.253 |
| aCO_2_ | NH_4_^+^ | 30 min | wounded | Df = 6 | *p* = 0.974 |
| aCO_2_ | NO_3_^-^ | 30 min | wounded | Df = 5 | *p* = 0.218 |
| aCO_2_ | NH_4_^+^ | 45 min | unwounded | Df = 6 | *p* = 0.685 |
| aCO_2_ | NO_3_^-^ | 45 min | unwounded | Df = 5 | *p* = 0.129 |
| aCO_2_ | NH_4_^+^ | 45 min | wounded | Df = 6 | *p* = 0.254 |
| aCO_2_ | NO_3_^-^ | 45 min | wounded | Df = 4 | *p* = 0.561 |
| eCO_2_ | NH_4_^+^ | 15 min | unwounded | Df = 6 | *p* = 0.824 |
| eCO_2_ | NO_3_^-^ | 15 min | unwounded | Df = 6 | *p* = 0.096 |
| eCO_2_ | NH_4_^+^ | 15 min | wounded | Df = 7 | *p* = 0.055 |
| eCO_2_ | NO_3_^-^ | 15 min | wounded | Df = 6 | *p* = 0.455 |
| eCO_2_ | NH_4_^+^ | 30 min | unwounded | Df = 6 | *p* = 0.347 |
| eCO_2_ | NO_3_^-^ | 30 min | unwounded | Df = 7 | *p* = 0.104 |
| eCO_2_ | NH_4_^+^ | 30 min | wounded | Df = 6 | *p* = 0.185 |
| eCO_2_ | NO_3_^-^ | 30 min | wounded | Df = 6 | *p* = 0.538 |
| eCO_2_ | NH_4_^+^ | 45 min | unwounded | Df = 6 | *p* = 0.069 |
| eCO_2_ | NO_3_^-^ | 45 min | unwounded | Df = 7 | *p* = 0.706 |
| eCO_2_ | NH_4_^+^ | 45 min | wounded | Df = 6 | *p* = 0.156 |
| eCO_2_ | NO_3_^-^ | 45 min | wounded | Df = 6 | *p* = 0.161 |
|  |  |  |  |  |  |
| **DHA (oxidized)** |  |  |  |  |  |
| aCO_2_ | NH_4_^+^ | 15 min | unwounded | Df = 6 | *p* = 0.809 |
| aCO_2_ | NO_3_^-^ | 15 min | unwounded | Df = 6 | *p* = 0.799 |
| aCO_2_ | NH_4_^+^ | 15 min | wounded | Df = 5 | *p* = 0.080 |
| aCO_2_ | NO_3_^-^ | 15 min | wounded | Df = 6 | *p* = 0.294 |
| aCO_2_ | NH_4_^+^ | 30 min | unwounded | Df = 7 | *p* = 0.224 |
| aCO_2_ | NO_3_^-^ | 30 min | unwounded | Df = 6 | *p* = 0.300 |
| aCO_2_ | NH_4_^+^ | 30 min | wounded | Df = 6 | *p* = 0.330 |
| aCO_2_ | NO_3_^-^ | 30 min | wounded | Df = 5 | *p* = 0.393 |
| aCO_2_ | NH_4_^+^ | 45 min | unwounded | Df = 6 | *p* = 0.378 |
| aCO_2_ | NO_3_^-^ | 45 min | unwounded | Df = 5 | *p* = 0.959 |
| aCO_2_ | NH_4_^+^ | 45 min | wounded | Df = 6 | *p* = 0.591 |
| aCO_2_ | NO_3_^-^ | 45 min | wounded | Df = 4 | *p* = 0.029 |
| eCO_2_ | NH_4_^+^ | 15 min | unwounded | Df = 6 | *p* = 0.716 |
| eCO_2_ | NO_3_^-^ | 15 min | unwounded | Df = 6 | *p* = 0.192 |
| eCO_2_ | NH_4_^+^ | 15 min | wounded | Df = 7 | *p* = 0.299 |
| eCO_2_ | NO_3_^-^ | 15 min | wounded | Df = 6 | *p* = 0.174 |
| eCO_2_ | NH_4_^+^ | 30 min | unwounded | Df = 6 | *p* = 0.643 |
| eCO_2_ | NO_3_^-^ | 30 min | unwounded | Df = 7 | *p* = 0.872 |
| eCO_2_ | NH_4_^+^ | 30 min | wounded | Df = 6 | *p* = 0.420 |
| eCO_2_ | NO_3_^-^ | 30 min | wounded | Df = 6 | *p* = 0.555 |
| eCO_2_ | NH_4_^+^ | 45 min | unwounded | Df = 6 | *p* = 0.121 |
| eCO_2_ | NO_3_^-^ | 45 min | unwounded | Df = 7 | *p* = 0.982 |
| eCO_2_ | NH_4_^+^ | 45 min | wounded | Df = 6 | *p* = 0.729 |
| eCO_2_ | NO_3_^-^ | 45 min | wounded | Df = 6 | *p* = 0.449 |
|  |  |  |  |  |  |
| **Total ascorbate** |  |  |  |  |  |
| aCO_2_ | NH_4_^+^ | 15 min | unwounded | Df = 6 | *p* = 0.446 |
| aCO_2_ | NO_3_^-^ | 15 min | unwounded | Df = 6 | *p* = 0.095 |
| aCO_2_ | NH_4_^+^ | 15 min | wounded | Df = 5 | *p* = 0.778 |
| aCO_2_ | NO_3_^-^ | 15 min | wounded | Df = 6 | *p* = 0.904 |
| aCO_2_ | NH_4_^+^ | 30 min | unwounded | Df = 7 | *p* = 0.080 |
| aCO_2_ | NO_3_^-^ | 30 min | unwounded | Df = 6 | *p* = 0.674 |
| aCO_2_ | NH_4_^+^ | 30 min | wounded | Df = 6 | *p* = 0.763 |
| aCO_2_ | NO_3_^-^ | 30 min | wounded | Df = 5 | *p* = 0.487 |
| aCO_2_ | NH_4_^+^ | 45 min | unwounded | Df = 6 | *p* = 0.431 |
| aCO_2_ | NO_3_^-^ | 45 min | unwounded | Df = 5 | *p* = 0.231 |
| aCO_2_ | NH_4_^+^ | 45 min | wounded | Df = 6 | *p* = 0.264 |
| aCO_2_ | NO_3_^-^ | 45 min | wounded | Df = 4 | *p* = 0.285 |
| eCO_2_ | NH_4_^+^ | 15 min | unwounded | Df = 6 | *p* = 0.927 |
| eCO_2_ | NO_3_^-^ | 15 min | unwounded | Df = 6 | *p* = 0.053 |
| eCO_2_ | NH_4_^+^ | 15 min | wounded | Df = 7 | *p* = 0.054 |
| eCO_2_ | NO_3_^-^ | 15 min | wounded | Df = 6 | *p* = 0.678 |
| eCO_2_ | NH_4_^+^ | 30 min | unwounded | Df = 6 | *p* = 0.340 |
| eCO_2_ | NO_3_^-^ | 30 min | unwounded | Df = 7 | *p* = 0.095 |
| eCO_2_ | NH_4_^+^ | 30 min | wounded | Df = 6 | *p* = 0.269 |
| eCO_2_ | NO_3_^-^ | 30 min | wounded | Df = 6 | *p* = 0.709 |
| eCO_2_ | NH_4_^+^ | 45 min | unwounded | Df = 6 | *p* = 0.1753 |
| eCO_2_ | NO_3_^-^ | 45 min | unwounded | Df = 7 | *p* = 0.537 |
| eCO_2_ | NH_4_^+^ | 45 min | wounded | Df = 6 | *p* = 0.202 |
| eCO_2_ | NO_3_^-^ | 45 min | wounded | Df = 6 | *p* = 0.479 |
|  |  |  |  |  |  |
| **Log_10_ DHA/Asc** |  |  |  |  |  |
| aCO_2_ | NH_4_^+^ | 15 min | unwounded | Df = 4 | *p* = 0.565 |
| aCO_2_ | NO_3_^-^ | 15 min | unwounded | Df = 6 | *p =* 0.405 |
| aCO_2_ | NH_4_^+^ | 15 min | wounded | Df = 5 | *p* = 0.08 |
| aCO_2_ | NO_3_^-^ | 15 min | wounded | Df = 6 | *p* = 0.116 |
| aCO_2_ | NH_4_^+^ | 30 min | unwounded | Df = 7 | *p* = 0.456 |
| aCO_2_ | NO_3_^-^ | 30 min | unwounded | Df = 6 | *p* = 0.129 |
| aCO_2_ | NH_4_^+^ | 30 min | wounded | Df = 6 | *p* = 0.391 |
| aCO_2_ | NO_3_^-^ | 30 min | wounded | Df = 5 | *p* = 0.089 |
| aCO_2_ | NH_4_^+^ | 45 min | unwounded | Df = 6 | *p* = 0.764 |
| aCO_2_ | NO_3_^-^ | 45 min | unwounded | Df = 5 | *p* = 0.747 |
| aCO_2_ | NH_4_^+^ | 45 min | wounded | Df = 6 | *p* = 0.349 |
| aCO_2_ | NO_3_^-^ | 45 min | wounded | Df = 4 | *p* = 0.178 |
| eCO_2_ | NH_4_^+^ | 15 min | unwounded | Df = 6 | *p* = 0.967 |
| eCO_2_ | NO_3_^-^ | 15 min | unwounded | Df = 6 | *p* = 0.491 |
| eCO_2_ | NH_4_^+^ | 15 min | wounded | Df = 7 | *p* = 0.588 |
| eCO_2_ | NO_3_^-^ | 15 min | wounded | Df = 6 | *p* = 0.244 |
| eCO_2_ | NH_4_^+^ | 30 min | unwounded | Df = 6 | *p* = 0.642 |
| eCO_2_ | NO_3_^-^ | 30 min | unwounded | Df = 7 | *p* = 0.895 |
| eCO_2_ | NH_4_^+^ | 30 min | wounded | Df = 6 | *p* = 0.224 |
| eCO_2_ | NO_3_^-^ | 30 min | wounded | Df = 6 | *p* = 0.446 |
| eCO_2_ | NH_4_^+^ | 45 min | unwounded | Df = 6 | *p* = 0.052 |
| eCO_2_ | NO_3_^-^ | 45 min | unwounded | Df = 5 | *p* = 0.808 |
| eCO_2_ | NH_4_^+^ | 45 min | wounded | Df = 6 | *p* = 0.961 |
| eCO_2_ | NO_3_^-^ | 45 min | wounded | Df = 6 | *p* = 0.395 |
|  |  |  |  |  |  |
| **OPDA** |  |  |  |  |  |
| aCO_2_ | NH_4_^+^ |  | unwounded | Df = 6 | *p* = 0.622 |
| aCO_2_ | NO_3_^-^ |  | wounded | Df = 6 | *p* = 0.649 |
| aCO_2_ | NH_4_^+^ |  | unwounded | Df = 6 | *p* = 0.821 |
| aCO2 | NO_3_^-^ |  | wounded | Df = 6 | *p* = 0.203 |
| eCO_2_ | NH_4_^+^ |  | unwounded | Df = 6 | *p* = 0.523 |
| eCO_2_ | NO_3_^-^ |  | wounded | Df = 6 | *p* = 0.438 |
| eCO_2_ | NH_4_^+^ |  | unwounded | Df = 6 | *p* = 0.574 |
| eCO_2_ | NO_3_^-^ |  | wounded | Df = 6 | *p* = 0.435 |
|  |  |  |  |  |  |
| **JA** |  |  |  |  |  |
| aCO_2_ | NH_4_^+^ |  | unwounded | Df = 6 | *p* = 0.795 |
| aCO_2_ | NO_3_^-^ |  | wounded | Df = 6 | *p* = 0.443 |
| aCO_2_ | NH_4_^+^ |  | unwounded | Df = 6 | *p* = 0.669 |
| aCO2 | NO_3_^-^ |  | wounded | Df = 6 | *p* = 0.391 |
| eCO_2_ | NH_4_^+^ |  | unwounded | Df = 6 | *p* = 0.059 |
| eCO_2_ | NO_3_^-^ |  | wounded | Df = 6 | *p* = 0.461 |
| eCO_2_ | NH_4_^+^ |  | unwounded | Df = 6 | *p* = 0.537 |
| eCO_2_ | NO_3_^-^ |  | wounded | Df = 6 | *p* = 0.558 |
|  |  |  |  |  |  |
| **JA-Ile** |  |  |  |  |  |
| aCO_2_ | NH_4_^+^ |  | unwounded | Df = 6 | *p* = 0.22 |
| aCO_2_ | NO_3_^-^ |  | wounded | Df = 6 | *p* = 0.385 |
| aCO_2_ | NH_4_^+^ |  | unwounded | Df = 6 | *p* = 0.793 |
| aCO2 | NO_3_^-^ |  | wounded | Df = 6 | *p* = 0.900 |
| eCO_2_ | NH_4_^+^ |  | unwounded | Df = 6 | *p* = 0.189 |
| eCO_2_ | NO_3_^-^ |  | wounded | Df = 6 | *p* = 0.459 |
| eCO_2_ | NH_4_^+^ |  | unwounded | Df = 6 | *p* = 0.225 |
| eCO_2_ | NO_3_^-^ |  | wounded | Df = 6 | *p* = 0.347 |
|  |  |  |  |  |  |
| **SA** |  |  |  |  |  |
| aCO_2_ | NH_4_^+^ |  | unwounded | Df = 6 | *p* = 0.284 |
| aCO_2_ | NO_3_^-^ |  | wounded | Df = 6 | *p* = 0.788 |
| aCO_2_ | NH_4_^+^ |  | unwounded | Df = 6 | *p* = 0.55 |
| aCO2 | NO_3_^-^ |  | wounded | Df = 6 | *p* = 0.759 |
| eCO_2_ | NH_4_^+^ |  | unwounded | Df = 6 | *p* = 0.922 |
| eCO_2_ | NO_3_^-^ |  | wounded | Df = 6 | *p* = 0.304 |
| eCO_2_ | NH_4_^+^ |  | unwounded | Df = 6 | *p* = 0.814 |
| eCO_2_ | NO_3_^-^ |  | wounded | Df = 6 | *p* = 0.667 |
|  |  |  |  |  |  |
| **ABA** |  |  |  |  |  |
| aCO_2_ | NH_4_^+^ |  | unwounded | Df = 6 | *p* = 0.369 |
| aCO_2_ | NO_3_^-^ |  | wounded | Df = 6 | *p* = 0.768 |
| aCO_2_ | NH_4_^+^ |  | unwounded | Df = 6 | *p* = 0.289 |
| aCO2 | NO_3_^-^ |  | wounded | Df = 6 | *p* = 0.632 |
| eCO_2_ | NH_4_^+^ |  | unwounded | Df = 6 | *p* = 0.08 |
| eCO_2_ | NO_3_^-^ |  | wounded | Df = 6 | *p* = 0.297 |
| eCO_2_ | NH_4_^+^ |  | unwounded | Df = 6 | *p* = 0.552 |
| eCO_2_ | NO_3_^-^ |  | wounded | Df = 6 | *p* = 0.588 |

**Abbreviations**: **ABA**: abscisic acid; **aCO_2_**: ambient carbon dioxide (450 ppm); **Asc:** reduced form of ascorbate; **C**:N: carbon-to-nitrogen; **DHA:** dehydroascorbate, oxidized form of ascorbate; **eCO_2_:** elevated carbon dioxide (900 ppm); **GSH**: reduced glutathione; **GSSG**: oxidized glutathione; **NAD:** oxidized form of nicotinamide adenine nucleotide; **NADH:** reduced form of nicotinamide adenine nucleotide; **NADP:** oxidized form of nicotinamide adenine nucleotide phosphate; **NADPH:** reduced form of nicotinamide adenine nucleotide phosphate; **NO_3_^-^**: nitrate; **NH_4_^+^**: ammonium ion; **JA**: jasmonic acid; **JA-Ile**: 7-jasmonyl-L-isoleucine; **OPDA**: 12-*oxo*-phytodienoic acid; **SA**: salicylic acid

**Literature cited**:

Armstrong, R.A. (2014) When to use the Bonferroni correction. *Ophthalmic &*

*Physiological Optics* 34, 502-508. Doi: 10.1111/opo.12131.
